# Treatment of methicillin-resistant *Staphylococcus aureus* (MRSA): updated guidelines from the UK

**DOI:** 10.1093/jacamr/dlaa114

**Published:** 2021-02-03

**Authors:** Nicholas M Brown, Anna L Goodman, Carolyne Horner, Abi Jenkins, Erwin M Brown

**Affiliations:** 1 Cambridge University Hospitals NHS Foundation Trust, Cambridge, UK; 2 Guy’s and St Thomas’ NHS Foundation Trust, London, UK; 3 MRC Clinical Trials Unit, University College London, London, UK; 4 British Society for Antimicrobial Chemotherapy, Birmingham, UK

## Abstract

These evidence-based guidelines are an updated version of those issued in 2008. They have been produced following a review of the published literature (2007–18) pertaining to the treatment of infections caused by MRSA. The guidelines update, where appropriate, previous recommendations, taking into account changes in the UK epidemiology of MRSA, ongoing national surveillance data and the efficacy of novel anti-staphylococcal agents licensed for use in the UK. Emerging therapies that have not been licensed for use in the UK at the time of the review have also been assessed.

## Contents

Executive Summary

Lay Summary

1. Introduction

2. The Working Party Report

2.1 What is The Working Party Report?

2.2 Why do we need a Working Party Report for these infections?

2.3 What is the purpose of the Report’s recommendations?

2.4 What is the scope of these guidelines?

2.5 What is the evidence for these guidelines?

2.6 Who developed these guidelines?

2.7 Who are these guidelines for?

2.8 How are the guidelines structured?

2.9 How frequently are the guidelines reviewed and updated?

2.10 Aim

3. Methodology

3.1 Evidence appraisal

3.2 Data sources and search strategy

3.3 Study eligibility and selection criteria

3.4 Data extraction and quality assessment

3.5 Rating of evidence and recommendations

3.6 Consultation process

4. Recommendations

4.1 Skin and soft tissue infections

4.1.1 Impetigo

4.1.2 Abscesses

4.1.3 Other skin and skin structure infections

4.2 Urinary tract infections

4.3 Bone and joint infections

4.4 Bacteraemia

4.5 Infective endocarditis

4.6 Respiratory tract infections

4.6.1 Necrotizing pneumonia

4.6.2 Nosocomial pneumonia

4.6.3 Ear, nose and throat or upper respiratory tract infections

4.7 Central nervous system and eye disease

4.7.1 Intracranial or spinal infections

4.7.2 Meningitis

4.7.3 Eye disease

5. Implementation of these guidelines

5.1 How can the guidelines be used to improve clinical effectiveness?

5.2 How much will implementation of the guidelines cost?

5.3 Summary of suggested audit measures

5.4 E-learning tools

6. Evidence gaps and further research

7. Conclusions

## Executive summary

Current UK guidelines for the treatment of MRSA are based on clinical evidence published more than 10 years ago.^1^ Much has changed since then, in particular, the incidence of MRSA in UK hospitals has fallen markedly since 2008.^2^ In addition, new anti-staphylococcal antibiotics have become available and experience of the use of these agents has increased. An update to the national MRSA treatment guideline in light of these changes is therefore required.

Updating the national guidelines relating to MRSA was a joint initiative of BSAC, British Infection Association (BIA), Healthcare Infection Society (HIS) and Infection Prevention Society (IPS). BSAC and BIA alone were involved in the production of this guideline. A separate guideline updating recommendations for infection prevention and control of MRSA will be developed by HIS and IPS.

The primary aim of this guideline was to update, where appropriate, previous recommendations, taking into account changes in the UK epidemiology of MRSA, ongoing national surveillance data and the efficacy of novel anti-staphylococcal agents licensed for use in the UK. Emerging therapies that had not been licensed for use in the UK at the time of the review were also included.

The most striking finding from the review process was the dearth of published evidence in this area. A total of 92 eligible articles were identified. After screening and review for eligibility, 30 studies were subsequently included in the guideline review. Many of these were non-inferiority studies performed for licensing purposes. Even though studies with a high risk of bias were excluded, others included too few patients with MRSA to be able to draw firm conclusions.

## Summary of recommendations

### Impetigo

- To prevent the development of antimicrobial resistance, consider an alternative to topical fusidic acid or mupirocin, for example a topical antiseptic such as hydrogen peroxide 1% cream, to treat impetigo caused by MRSA where there is localized, non-bullous disease and the patient is clinically well. Consider topical fusidic acid or mupirocin as a second-line option in this clinical setting and only when the MRSA isolate is known to be susceptible (weak recommendation).
- Treat complicated impetigo using systemic antimicrobial therapy with the choice of agent determined by susceptibility testing (strong recommendation).

### Abscesses

- Use incision and drainage to treat abscesses caused by MRSA (strong recommendation).
- Do not use antibiotics routinely in patients with abscesses caused by MRSA that are drained, are less than 5 cm in diameter, and where there is no systemic response (fever and/or cellulitis) and/or immunodeficiency, including neutropenia and defects of cell-mediated immunity (strong recommendation).
- Use antibiotics in combination with incision and drainage in patients with abscesses caused by MRSA PFGE strain type USA300, or where this is likely to be the most prevalent strain (strong recommendation).
- Use oral clindamycin or co-trimoxazole when oral treatment is warranted, and the MRSA isolate is known to be susceptible (strong recommendation).

### Other skin and skin structure infections

- For severe cellulitis/soft tissue infection caused by MRSA use intravenous glycopeptides (vancomycin or teicoplanin) (strong recommendation).
- Use linezolid (oral or intravenous) or daptomycin (intravenous) as an alternative (strong recommendation).
- Consider tigecycline as an alternative when first- and second-line agents are contraindicated, and the isolate is susceptible (weak recommendation).
- Consider clindamycin, co-trimoxazole, or doxycycline as oral agents (when the isolate is susceptible) for treatment of patients with mild skin and soft tissue infection caused by MRSA, or for oral stepdown therapy (weak recommendation).
- Consider recently licensed agents such as ceftaroline, delafloxacin, oritavancin, or telavancin as alternative options for treatment of cellulitis/soft tissue infection caused by MRSA (weak recommendation).
- No recommendations can be made on the use of ceftobiprole, dalbavancin and tedizolid over standard therapeutic agents in the treatment of SSTI caused by MRSA.

### Urinary tract infection (UTI)

- Exclude the presence of MRSA bacteraemia before commencing treatment of MRSA isolated from urine (weak recommendation).
- Consider treating a genuine lower UTI caused by MRSA with an oral agent, such as doxycycline, trimethoprim, ciprofloxacin, or co-trimoxazole, according to susceptibility (weak recommendation).
- For complicated UTI caused by MRSA consider intravenous glycopeptides (vancomycin or teicoplanin) as the first-line treatment (weak recommendation).
- When a glycopeptide is contraindicated consider daptomycin as an alternative agent if intravenous therapy is required (weak recommendation).
- Linezolid is not recommended for the treatment of MRSA UTI, given its poor excretion by the kidney (weak recommendation).
- For catheter-associated UTI caused by MRSA, whenever possible/practicable replace the catheter, with or without a single dose of gentamicin if the MRSA isolate is known to be susceptible (weak recommendation). Consider a single dose of glycopeptide (vancomycin or teicoplanin) as an alternative if the isolate is resistant to gentamicin or there are other contraindications (weak recommendation).

### Bone and joint infections

- Use a multidisciplinary approach for treatment of MRSA bone and joint infections, including surgery or drainage where indicated (strong recommendation).
- For bone and joint infections caused by MRSA use intravenous glycopeptides (vancomycin or teicoplanin) as the first-line choice of treatment (strong recommendation).
- Consider 2 weeks of intravenous glycopeptide (vancomycin or teicoplanin) followed by further intravenous or oral antibiotics to complete a total treatment course of a minimum of 4 weeks for septic arthritis or 6 weeks for osteomyelitis (weak recommendation).
- Use therapeutic drug monitoring to ensure that non-toxic, therapeutic pre-dose serum concentrations of 15–20 mg/L for vancomycin, or 20–40 mg/L for teicoplanin are achieved (strong recommendation).
- When a glycopeptide is contraindicated consider daptomycin (6 mg/kg dose) or linezolid as alternative agents (weak recommendation).
- Use clindamycin, co-trimoxazole, doxycycline, or linezolid as oral options to complete treatment when the MRSA isolate is known to be susceptible (strong recommendation).
- Do not use rifampicin, fusidic acid or a quinolone as a single oral agent; use in combination with other agents to which the isolate is susceptible (strong recommendation).

### Bacteraemia

- Use intravenous vancomycin for uncomplicated bacteraemia caused by MRSA (strong recommendation).
- When vancomycin is contraindicated use linezolid as an alternative first-line choice of treatment (strong recommendation).
- When first-line agents are contraindicated consider daptomycin or teicoplanin (weak recommendation).
- Do not use co-trimoxazole alone as a first-line agent for MRSA bacteraemia, however, consider using it as an oral step-down when the MRSA isolate is known to be susceptible (weak recommendation).
- Consider a minimum duration of 14 days of antibiotic therapy for uncomplicated bacteraemia and a minimum duration of 28 days for complicated bacteraemia caused by MRSA (weak recommendation).

### Endocarditis

Refer to the most recent version of the BSAC endocarditis treatment guideline.

### Necrotizing pneumonia

- For necrotizing pneumonia caused by MRSA use intravenous vancomycin or linezolid (strong recommendation).
- Consider addition of a toxin-inhibiting agent, such as clindamycin or rifampicin, when the MRSA isolate is known to be susceptible (weak recommendation).

### Nosocomial pneumonia

- In the absence of at least one additional randomized controlled trial (RCT) confirming the superiority of linezolid over vancomycin for nosocomial pneumonia caused by MRSA, ideally associated with a low risk of bias, we have opted to recommend either intravenous vancomycin or linezolid as first-line therapy (weak recommendation).
- Do not use daptomycin to treat nosocomial pneumonia caused by MRSA as it is inactivated by lung surfactant (strong recommendation).
- No recommendations can be made on the use of ceftobiprole over standard therapeutic agents in the treatment of HAP caused by MRSA.

### Ear, nose and throat or upper respiratory tract infection

- For severe MRSA-associated ear, nose and throat or upper respiratory tract infections consider intravenous glycopeptide (vancomycin or teicoplanin) or linezolid (weak recommendation).
- For minor/less-severe infections consider co-trimoxazole or doxycycline as an oral option when the MRSA isolate is known to be susceptible (weak recommendation).

### Intracranial or spinal infection

- Whenever clinically possible, source control is necessary for intracranial and spinal infections (strong recommendation).
- Unless surgical intervention is contraindicated use incision and drainage for treatment of intracranial and spinal infections caused by MRSA (strong recommendation).
- In the absence of neurological deficits consider treating small epidural abscesses with antibiotics alone (weak recommendation).
- For treatment of intracranial and spinal infections caused by MRSA consider intravenous vancomycin or linezolid as the first-line choice of treatment (weak recommendation).

### Meningitis

- For meningitis caused by MRSA use intravenous vancomycin (strong recommendation). For severe infection, consider adding rifampicin according to susceptibility (weak recommendation).
- Use therapeutic drug monitoring to ensure that non-toxic, therapeutic pre-dose serum concentrations (15–20 mg/L) of vancomycin are achieved (strong recommendation).
- In severe cases, or when the patient fails to respond to intravenous vancomycin, patients should be transferred to a neurosurgical centre for instillation of vancomycin directly into the ventricles (strong recommendation).
- Do not use clindamycin, chloramphenicol or linezolid to treat meningitis caused by MRSA (strong recommendation). These drugs are not bactericidal, such activity being a requirement of antibiotics used as therapy of patients with meningitis.
- No recommendation can be made for the use of teicoplanin in this clinical setting.

### Eye infection

- For superficial MRSA eye disease consider gentamicin or chloramphenicol eye drops according to isolate susceptibility (weak recommendation).
- Consider dissemination secondary to bacteraemia when a patient is diagnosed with endophthalmitis caused by MRSA (strong recommendation).
- For deep-seated eye infections caused by MRSA consider a multidisciplinary approach comprising specialist ophthalmologists and infection specialists (weak recommendation).
- For deep-seated eye infections caused by MRSA consider intravitreal vancomycin and systemic quinolones according to susceptibility (weak recommendation).
- Consider oral linezolid as a treatment option, recognizing that there is limited evidence of efficacy in MRSA infection at this site (weak recommendation).

## Lay summary

‘MRSA’ stands for methicillin-resistant *Staphylococcus aureus*, which is a type of bacteria that can cause infection. Infection with MRSA mainly occurs in people who are already ill and can occur wherever healthcare is given. This can be in hospital or in the community setting, such as in care homes, nursing homes or at home. Options to treat MRSA infection are sometimes limited because MRSA are resistant to a particular group of antibiotics (penicillins) that would commonly be used to treat *Staphylococcus aureus* infections. This means the bacteria are unaffected by penicillins, and the patient is unlikely to respond to treatment with this group of antibiotics.

The number of MRSA bloodstream infections in UK hospitals has fallen since 2008, which affects how patients with sepsis, a serious life-threatening infection, are treated. Antibiotics are one of the main treatments for sepsis. Identifying the most appropriate antibiotic and giving it promptly increases the possibility of surviving sepsis, including in patients who may have MRSA.

There is a broad range of antibiotics available to treat patients with infections caused by MRSA, and new ones have become available since the last guideline was published, but we still need evidence to find out the benefits of these new antibiotics. This is particularly relevant to the treatment of patients with some deep-seated or difficult-to-treat infections caused by MRSA, such as bone and joint infections, where antibiotics are often given for long periods, which can result in more side effects for patients.

This guideline is intended to help the clinical care of patients with suspected or confirmed MRSA infection in the UK. The guidelines may also be of use to patients with an MRSA infection, those who care for patients with an MRSA infection, and the general public, by helping them to understand which treatments may be an appropriate option for them.

## 1. Introduction

Current UK guidelines for the treatment of MRSA are based on clinical evidence published more than 10 years ago.^1^ Much has changed since then, including observed changes in the nature, incidence and epidemiology of MRSA infections. In particular, the incidence of MRSA in UK hospitals has fallen markedly since 2008.^2^ Also, for reasons that are unclear, community strains of MRSA, such as PFGE strain-type USA300, have not become established in the UK, despite frequent introductions.^3^

Unlike infections caused by antibiotic-resistant aerobic Gram-negative bacteria, there is a broad range of antibiotics available to treat patients with infections caused by MRSA. The clinical usage of agents such as linezolid and daptomycin was limited when the previous MRSA guidelines were published;^1^ however, during the intervening period other agents have been licensed or are close to being licensed and their places in therapeutic guidelines are unclear. This is particularly relevant to the treatment of patients with some deep-seated or difficult-to-treat infections caused by MRSA, such as bone and joint infections, where the durations of therapy are prolonged and the morbidity remains high.

## 2. The Working Party report

### 2.1 What is the Working Party report?

The report is a set of recommendations covering key aspects of MRSA treatment in a range of specific infections. The guidelines review the evidence published since the last UK MRSA treatment guidelines were published in 2008.^1^ The prevention of MRSA infection is not included in these guidelines. The Working Party recommendations have been developed systematically through multi-disciplinary discussions based on published evidence. They should be used in the development of local protocols for all relevant healthcare settings.

### 2.2 Why do we need a Working Party report for these infections?

The clinical picture of MRSA infection has changed significantly in the 10 years since the previous review. In addition, new antibiotics have become available and experience of the use of these has increased. An update to the national treatment and management guideline in light of these changes is therefore required.

### 2.3 What is the purpose of the report’s recommendations?

The objectives of the guideline review can be summarized as follows: (i) to improve the quality of care provided to patients (children and adults) with MRSA infection; (ii) to provide an educational resource for all relevant healthcare professionals; (iii) to encourage a multidisciplinary approach to the management of MRSA infection; and (iv) to promote a standardized approach to the management of MRSA infection.

### 2.4 What is the scope of these guidelines?

This guideline is intended to assist in the clinical care of patients with suspected or confirmed MRSA infection in the UK. The 2008 MRSA guideline addressed both prophylaxis and treatment of MRSA; however, prophylaxis has not been included in the current guideline. Recommendations relating to infection prevention and control of MRSA, including decolonization, are considered in a separate guideline written by HIS and IPS. Although some guidance may be given as to dosing or drug interactions in particular cases, this was not considered to be within the aim of these guidelines and users should seek information on dosing and interactions elsewhere, such as in the British National Formulary (BNF).

### 2.5 What is the evidence for these guidelines?

To prepare these recommendations, the Working Party derived questions for review and collectively reviewed relevant peer-reviewed research. Methods are described fully below; they were in accordance with National Institute for Health and Care Excellence (NICE) principles and the Cochrane handbook for systematic reviews of interventions.^4^

### 2.6 Who developed these guidelines?

Updating the national guidelines relating to MRSA was a joint initiative of BSAC, BIA, HIS and IPS. BSAC and BIA alone were involved in the production of this guideline. HIS and IPS are responsible for updating recommendations for infection prevention and control of MRSA, which will be available in a separate guideline. The guideline was reviewed independently by two lay representatives.

The Working Party comprised infectious diseases and microbiology clinicians, a pharmacist and a clinical scientist. Two lay representatives prepared the lay summary and contributed additional comments to the guideline.

The views expressed in this publication are those of the authors and have been endorsed by BSAC and BIA following consultation.

### 2.7 Who are these guidelines for?

Any healthcare practitioner may use these guidelines and adapt them for their use. It is anticipated that users will include clinical staff. It is expected that these guidelines will also raise awareness of MRSA and the complexities of its treatment amongst clinicians who care for patients with infections. The guideline may also be read by patients with MRSA infection, helping them to understand which treatments may be appropriate options for them.

### 2.8 How are the guidelines structured?

Each section comprises background information, a summary of the evidence base with levels, and a recommendation graded according to the available evidence.

### 2.9 How frequently are the guidelines reviewed and updated?

The guideline will be reviewed at least every 4 years and updated if changes in the evidence are sufficient to require a change in practice.

### 2.10 Aim

The primary aim of this report was to update, where appropriate, previous recommendations, taking into account changes in the UK epidemiology of MRSA, ongoing national surveillance data, and the efficacy of novel anti-staphylococcal agents licensed for use in the UK. Emerging therapies that have not been licensed for use in the UK at the time of the review have also been assessed.

## 3. Methodology

### 3.1 Evidence appraisal

Questions to guide evidence review were designed according to PICO principles^4^ and agreed by the Working Party. The overall question was what new evidence had become available to support treatment options in the management of MRSA since the last literature review and published guideline in 2008.^1^

The *Population* considered by the guideline was patients infected with MRSA. The conditions assessed were based on the previous MRSA guideline and as such included: impetigo; abscesses; other skin and skin structure infections; urinary tract infections; bone and joint infections; bacteraemia; infective endocarditis; necrotizing pneumonia; nosocomial pneumonia; ear, nose and throat and upper respiratory tract infections; intracranial or spinal infections; cerebrospinal fluid infections; and eye disease. The *Interventions* assessed by the guideline were antimicrobial agents evaluated by studies since the last guideline in 2008. The *Comparisons* used were as per study authors (likely to be standards of care, such as vancomycin or placebo). The primary *Outcome* measure, clinical and/or microbiological cure, was assessed according to that stated within each individual study design. When adverse events were reported these were noted but were not assessed as an outcome measure due to inconsistency of reporting between studies.

### 3.2 Data sources and search strategy

The Cochrane Library (including the Central Register of Controlled Trials), EMBASE, and MEDLINE databases were comprehensively searched from 1 January 2006 to 26 March 2017 (Table S1, available as Supplementary data at *JAC-AMR* Online). A further search using the same criteria, but covering the period 1 January 2016 to 31 August 2018, was undertaken in order to identify any additional papers published since the initial search.

### 3.3 Study eligibility and selection criteria

Only articles that contained original, relevant and interpretable data about the management of MRSA infection and which were published in full in peer-reviewed English language journals were acceptable. The following study designs were eligible for inclusion: randomised control trials (RCTs), controlled clinical trials (CCTs), interrupted time series (ITS) with at least three data points before and after implementation of the intervention and controlled before and after studies (CBAs) that were undertaken in three or more centres/hospitals. Exclusion criteria were studies that did not have comparator groups, studies in which the results from multiple studies with different designs were pooled and studies in which results for MRSA were not specified (Table S2). The Cochrane Collaboration’s tool for assessing risk of bias in randomized trials was used to assess the quality of studies.^5^ In the absence of other sources of bias, studies with low numbers of participants (<10) in each treatment arm were considered to have an unacceptable risk of bias and were excluded outright. Studies with between 10–49 participants in each treatment arm were considered to have a high risk of bias and were reviewed on an individual basis. Studies with between 50–199 patients in each treatment arm were considered to have a moderate risk of bias, whereas studies with >200 patients in each treatment arm were considered to have low risk of bias.^6^ The risk of bias was reflected in the grading of the evidence for each study included. Systematic reviews/meta-analyses were excluded, but their bibliographies were cross checked in order to identify studies not captured by the current literature searches. Characteristics of the studies that met the inclusion criteria are summarized in Table S3. When adverse events were reported these were noted but were not assessed as an outcome measure due to inconsistency of reporting between studies.

### 3.4 Data extraction and quality assessment

**Figure 1.**
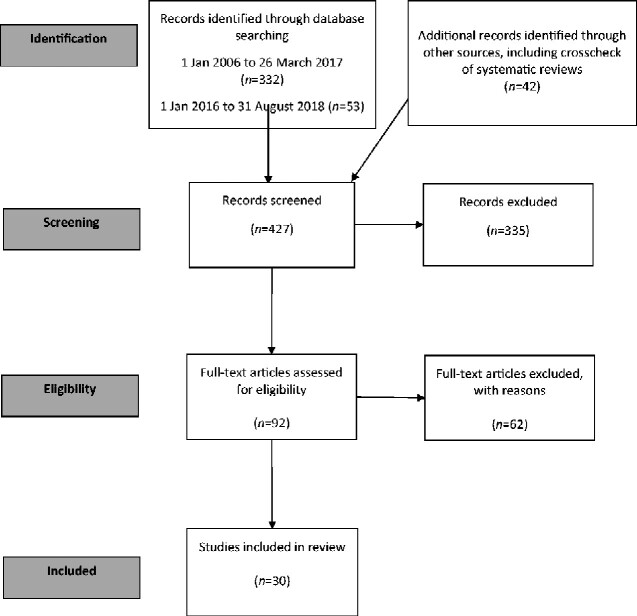
Flow diagram illustrating stages of the literature search and systematic review.^51^

A total of 332 eligible articles was identified from the first literature search and 53 references in the second search (Figure 1). The abstracts of all articles identified by the literature searches were screened by two reviewers for clinical trials concerned with the treatment of patients with infections caused by MRSA that had been published as full papers in peer-review journals: any differences were resolved by discussion and consensus. The full papers of studies meeting these criteria were obtained and they were assessed by both reviewers, principally in terms of design criteria; again, any differences were resolved by discussion and consensus. In the event of uncertainty or failure to agree, studies were referred to the guideline development group. Studies identified as being eligible for further consideration were referred to members of the guideline development group who determined whether they should be included or excluded and independently performed data extraction on the included studies. The full papers of all studies which were deemed eligible for inclusion were reviewed in order to identify those that fulfilled the criteria for inclusion; reasons for exclusion were recorded (Table S2, which lists the excluded studies and reasons for their exclusion). Two review authors independently performed data extraction from the included studies (Table S3) recording information on study design, type of intervention, presence of controls, type of targeted behaviour, participants, setting, methods (unit of allocation, unit of analysis, study power), primary and secondary outcome measures and results.

### 3.5 Rating of evidence and recommendations

This guideline was developed in accordance with the AGREE II instrument.^7^ The rating of evidence was modified from that used by SIGN50^8^ to be relevant to the study types included in this guideline. The strength of recommendation was adopted from GRADE (Grades of Recommendations Assessment, Development and Evaluation) (Table 1).^9^

**Table 1.** Levels of evidence for intervention studies (A) (modified from^8^) and grading of recommendations (B)^9^

A	Levels of Evidence
1++	Two or more RCTs with a very low risk of bias.
1+	Two or more RCTs with a low risk of bias.
1−^a^	One or more RCTs with a high risk of bias.
2++	Interrupted time series with a control group: (i) there is a clearly defined point in time when the intervention occurred; and (ii) at least three data points before and three data points after the intervention.
2+	Controlled before–after studies with two or more intervention and control sites.
2−^a^	Interrupted time series without a parallel control group: (i) There is a clearly defined point in time when the intervention occurred; and (ii) at least three data points before and three data points after the intervention. Controlled before–after studies with one intervention and one control site.
3	Non-analytic studies (e.g. uncontrolled before–after studies).
4	Expert opinion. Legislation.

B	Grading of Recommendations

For ‘strong’ recommendations: When the desirable effects of an intervention clearly outweigh the undesirable effects. When the desirable effects of an intervention clearly *do not* outweigh the undesirable effects. For ‘weak’ recommendations: When the desirable effects probably outweigh undesirable effects. When the undesirable effects probably outweigh desirable effects.	

Key: RCT, randomized controlled trial.

a Studies with an evidence level of 1− and 2− were not used on their own as a basis for making a recommendation.

Based on the analysis of the results, changes to previous recommendations were made and new recommendations were proposed. Previous guidelines were used for comparison of the evidence identified in this review.^1^^,^^10^ Some recommendations were updated for pragmatic reasons in the absence of new evidence to provide improved clarity to the reader in particular clinical situations, such as MRSA meningitis.

### 3.6 Consultation process

Draft recommendations were written by the guideline development group. These were circulated (on 6 January 2020) to a comprehensive list of stakeholders (Table S4) and uploaded to the BSAC website (www.bsac.org.uk) for a 5 week consultation period. Final alterations were made to the document in response to the consultation process (Table S5).

## 4. Recommendations

### 4.1 Skin and soft tissue infections

Acute bacterial skin and soft tissue infection (SSTI) was the most common category of infection in the new studies reviewed. Many of the studies were industry-sponsored, performed for licensing purposes and powered to demonstrate non-inferiority in comparison with current standard treatment, which was most often vancomycin. There was marked heterogeneity in the types of infections studied. Several studies enrolled patients with infections caused by bacteria other than just MRSA. Several of the excluded studies could not be assessed due to the small number of patients with proven MRSA infection.

In 2013 FDA guidance for the conduct of studies involving patients with acute bacterial skin and skin structure infections (ABSSSI) was updated.^11^ The new FDA guidance sought to provide more consistency in study design and recommended that the primary efficacy endpoint in trials should be measured at 48–72 h, rather than at the end of therapy or at post-treatment review.^11^ Included studies have varied in their compliance with this, according to their date of publication.

#### 4.1.1 Impetigo

##### Evidence

Older evidence in this area is reviewed in the PHE primary care guidance,^12^ a Cochrane Review,^13^ and NICE Clinical Knowledge Summaries (CKS): Impetigo (2015).^14^ Uncomplicated impetigo, defined as localized non-bullous impetigo in an immunocompetent individual with no systemic signs of infection, may respond to topical antiseptics, such as hydrogen peroxide 1% cream.^14^ The guideline development group is concerned about the use of topical fusidic acid and mupirocin owing to evidence of the emergence of resistant strains following their application, notwithstanding their availability as treatment options with proven efficacy. More extensive or complicated impetigo may require systemic antibiotic therapy, but no new evidence relating to the optimal agent(s) specifically for infection caused by MRSA was identified.

Only one new study was identified regarding the treatment of impetigo caused by MRSA.^15^ Topical retapamulin, an agent of the pleuromutilin class, has recently been licensed for the treatment of impetigo. Retapamulin ointment 1% administered twice daily for 5 days was shown to be inferior to oral treatment with linezolid 600 mg 12 hourly for 10 days (or equivalent dose adjusted for age in children) in an RCT including adults and children with either infected wounds or impetigo caused by MRSA.^15^ The primary outcome measure was clinical cure at follow up 7–9 days after the completion of therapy; 410 patients were enrolled and 125 of these had impetigo. Across the intention-to-treat study population infected with MRSA, the clinical cure rate in the retapamulin group [41/72 (56.9%)], was significantly lower than that in the oral linezolid group [32/38 (84.2%); difference −27.3%; 95% CI −45.8% to −8.7%]. However, outcome data were not provided for the subset of patients with impetigo [79 in the retapamulin arm versus 46 in the linezolid arm (all cause)]. The absence of this information, together with the small number of patients in each group (with the associated moderate-to-high risk of bias), precludes reaching conclusions regarding the efficacy of this novel agent as treatment of impetigo specifically.

##### Quality of the evidence

No new evidence regarding the treatment of impetigo caused by MRSA was identified in the current review.

##### Recommendations

- To prevent the development of antimicrobial resistance, consider an alternative to topical fusidic acid or mupirocin, for example a topical antiseptic such as hydrogen peroxide 1% cream, to treat impetigo caused by MRSA where there is localized, non-bullous disease and the patient is clinically well. Consider topical fusidic acid or mupirocin as a second-line option in this clinical setting and only when the MRSA isolate is known to be susceptible (weak recommendation).
- Treat complicated impetigo using systemic antimicrobial therapy, with the choice of agent determined by susceptibility testing (strong recommendation).

#### 4.1.2 Abscesses

##### Evidence

There is robust evidence that antibiotic treatment is not required routinely in patients with uncomplicated abscesses caused by MSSA who undergo incision and drainage, provided there is no evidence of a systemic inflammatory response (fever or cellulitis) or immunodeficiency.^16^ Hitherto, it has been assumed that the same principles apply to abscesses caused by MRSA.

Five new studies including patients with abscesses were identified during the present systematic review.^17–21^ In each of these studies, patients with abscesses due to a range of infective organisms were included, but there were sufficient patients with MRSA in each arm of the studies to allow evaluation.

Chen *et al.* (2011)^17^ compared the use of oral clindamycin or cefalexin (an antimicrobial agent with no activity against MRSA), in an RCT in 200 children with uncomplicated abscesses presenting to an emergency department. The abscesses were drained (either spontaneously or surgically), but hospital admission was not required. Children with impaired immunity were excluded. The majority of wound cultures (69%) grew MRSA, and the majority of isolates (93%) were Panton Valentine Leucocidin (PVL)-positive PFGE strain type USA300 strains; 91% of MRSA isolates were clindamycin susceptible. Success was high at both the primary endpoint (improvement at 48–72 h) and secondary outcome measure (resolution of infection at 7 days). For the subgroup of patients with MRSA, 6/64 (9%) patients in the cefalexin arm and 2/71 (3%) in the clindamycin arm were reported to have deteriorated at the 48–72 h visit. This difference was not statistically significant (*P = *0.15). Among patients who could be evaluated at 7 days, cure was reported in 63/63 (100%) patients in the cefalexin arm and 66/70 (94%) patients in the clindamycin arm (*P = *0.12). Although, in this study, there was no evidence that antibiotics were beneficial, the small number of patients, with resultant moderate risk of bias, precludes making reliable conclusions.

Holmes *et al.* (2016)^19^ conducted an RCT powered for non-inferiority comparing oral co-trimoxazole given for 3 days or the same drug given for 10 days in immunocompetent children with abscesses requiring drainage presenting to an emergency department in the US. The primary outcome measure was cure as assessed at a 10–14 day follow-up visit. Of the 265 children recruited, MRSA was isolated from abscess cultures from 69 in each arm; all the MRSA isolates were susceptible to co-trimoxazole. Overall, there was no statistically significant difference in outcome between the two groups at the primary endpoint. However, in the subset of patients with MRSA, the treatment failure rate was 8/69 (12%) in the 3 day treatment arm versus 1/69 (1%) in the 10 day arm (*P = *0.03). This failed to meet the predefined criteria for non-inferiority, which was a difference of no more than 7%. In any event, the small number of patients, with the associated moderate risk of bias, precludes drawing reliable conclusions.

A large multicentre RCT included both adults and children in the US presenting with small skin abscesses (≤5 cm in diameter).^18^ All patients underwent incision and drainage and were then randomized to receive either clindamycin (300 mg 8 hourly for adults), co-trimoxazole (480 mg twice daily for adults) or placebo for 10 days; appropriate dose adjustment was made for children. The primary outcome measure was clinical cure at the end of treatment. A total of 786 patients were enrolled and MRSA was isolated from cultures in 388 of these. The overall clinical cure rate in the evaluable population was statistically significantly higher in patients treated with antibiotics [clindamycin 221/238 (92.9%), co-trimoxazole 215/232 (92.7%)], compared with those who received the placebo [177/220 (80.5%)] (*P < *0.001). This was also the case in patients infected with MRSA [clindamycin 116/126 (92.1%), co-trimoxazole 110/117 (94%), placebo 73/96 (76%)] (*P < *0.001). There was no statistically significant difference in cure rates between patients treated with clindamycin or co-trimoxazole. However, the small number of patients with MRSA in each group (fewer than 200), which resulted in a moderate risk of bias, precludes drawing reliable conclusions. In subset analysis the benefit of antibiotic treatment was seen with infections caused by MSSA or MRSA, but not with infections where no *S. aureus* was isolated. These findings challenge the view that antibiotics offer no benefit over incision and drainage in small abscesses caused by *S. aureus*.

Talan *et al*. (2016)^21^ performed a similar large multicentre RCT in patients over 12 years of age in the US presenting with an uncomplicated abscess and treated as outpatients following drainage. Patients were randomized to receive either high-dose oral co-trimoxazole (1920 mg twice daily) or placebo for 7 days; 1265 patients were recruited and MRSA was isolated from specimens obtained following drainage from 565 [394 of 410 MRSA isolates tested (96.1%) were PFGE strain type USA300]. Overall clinical cure rates in the per protocol population at the primary endpoint, the assessment at the end of treatment, demonstrated an advantage of antibiotic therapy over placebo [co-trimoxazole 487/524 (92.9%) versus placebo 457/533 (85.7%)] (*P < *0.001). However, this difference was not observed at the FDA guidance early endpoint assessment after 48-72 h of treatment. Results were not presented for the subgroup of patients with MRSA; however, Talan *et al.* (2018)^20^ subsequently performed further analysis of the data from this study. In the per protocol population with infection caused by MRSA the response rate following treatment with co-trimoxazole was statistically significantly higher than that with placebo [203/219 (92.7%) versus 202/249 (81.1%)] (difference 11.6%; 95% CI 5.2%–18.0%). In patients with MSSA, a smaller statistically non-significant benefit was seen [co-trimoxazole 78/86 (90.7%) versus placebo 71/86 (82.6%)] (difference 8.1%; 95% CI −3.1% to +19.4%), whereas in patients from whom neither MRSA nor MSSA was recovered, there was no benefit of antibiotic [co-trimoxazole 203/216 (94%) versus placebo 181/195 (92.8%)] (difference 1.2%; 95% CI −4.1% to +6.5%). This suggests that antibiotic treatment of abscesses after incision and drainage may be of benefit in the subset of patients with infection caused by MRSA.

The aforementioned studies were performed in the US in a setting where PVL-positive PFGE strain type USA300 was the predominant MRSA strain in circulation. This is not the case in the UK and therefore it is unclear if the findings can be extrapolated to the UK, where the incidence of community strains of MRSA, especially USA300, is low. A UK guideline for management of community-acquired MRSA published in 2010 recommended that immunocompetent patients with uncomplicated abscesses (less than 5 cm in diameter) without cellulitis do not require antibiotic therapy after drainage.^10^

##### Quality of the evidence

Incision and drainage of abscesses is necessary for a successful outcome (quality of evidence: 1+); however, the addition of antibiotics is not always necessary (quality of evidence: 1++).

Evidence from geographical areas where MRSA PFGE strain type USA300 is common supports the use of antibiotics in combination with incision and drainage (quality of evidence: 1+).

In circumstances when antibiotic treatment is necessary, there is evidence that clindamycin or co-trimoxazole may be suitable oral agents if the isolate is susceptible (quality of evidence: 1+).

##### Recommendations

- Use incision and drainage to treat abscesses caused by MRSA (strong recommendation).
- Do not use antibiotics routinely in patients with abscesses caused by MRSA that are drained, are less than 5 cm in diameter, and where there is no systemic response (fever and/or cellulitis) and/or immunodeficiency, including neutropenia and defects of cell-mediated immunity (strong recommendation).
- Use antibiotics in combination with incision and drainage in patients with abscesses caused by MRSA PFGE strain type USA300, or where this is likely to be the most prevalent strain (strong recommendation).
- Use oral clindamycin or co-trimoxazole when oral treatment is warranted, and the MRSA isolate is known to be susceptible (strong recommendation).

#### 4.1.3 Other skin and skin structure infections

##### Evidence

Glycopeptides are the current standard of care for the initial therapy of cellulitis caused by MRSA. Since the previous guideline was published, linezolid and daptomycin have become established alternatives, particularly in hospitalized patients, although oral linezolid and intravenous daptomycin are frequently used to facilitate discharge from hospital [the latter in the outpatient parenteral antimicrobial therapy (OPAT) setting].

Additional evidence which supports the use of linezolid as treatment of patients with SSTI caused by MRSA was identified by the current systematic review. One study meeting the guideline inclusion criteria demonstrated that linezolid was not inferior to vancomycin.^22^ In this open-label randomized case-control study, patients with suspected MRSA infection were randomized to receive linezolid 600 mg twice daily (given either intravenously or orally) or intravenous vancomycin 15 mg/kg twice daily (dose adjusted according to therapeutic drug monitoring) for 7–14 days. The primary outcome measure was clinical success in the per protocol population at the end of the study and was achieved in 191/227 (84%) patients in the linezolid arm and 167/209 (80%) patients in the vancomycin arm (*P = *0.249).

No new studies comparing daptomycin with vancomycin and fulfilling the inclusion criteria were identified in the current systematic review.

Talan *et al.* (2016)^23^ performed a RCT comparing clindamycin 300 mg 6 hourly with co-trimoxazole 1920 mg 12 hourly in patients >12 years of age presenting to emergency departments in the USA with uncomplicated wound infections. Treatment was administered for 7 days. The primary outcome measure was clinical cure as assessed at 7–14 days. The study was powered to demonstrate superiority of clindamycin over co-trimoxazole and this required the lower value of the 95% CI of the difference in clinical cure to be greater than zero. Overall, 500 patients were enrolled and 161 patients with proven MRSA infection were included in the per protocol population. There were no statistically significant differences between the treatment arms and clinical cure was seen in 70/78 (89.7%) patients treated with clindamycin and 78/83 (94%) of patients treated with co-trimoxazole (difference −4.2%; 95% CI −13.9% to +5.5%). Given that there were fewer than 100 patients in each arm, there would be a moderate risk of bias, thereby undermining the reliability of the findings.

Several studies evaluated new agents with activity against MRSA as treatment for patients with skin infections. These include: the new anti-staphylococcal cephalosporins, ceftaroline^24^ and ceftobiprole;^25^^,^^26^ new glycopeptides, dalbavancin,^27^ oritavancin,^28–30^ and telavancin;^31^ a new oxazolidinone, tedizolid;^32^^,^^33^ two pleuromutilin antibiotics, lefamulin^34^ and retapamulin;^15^ a new quinolone, delafloxacin;^35–37^and iclaprim.^38^^,^^39^ At the time of writing, ceftobiprole, lefamulin and iclaprim had not been licensed for this clinical indication. No studies meeting the guideline inclusion criteria assessed the efficacy of omadacycline in this clinical setting.

Cephalosporins with enhanced activity against Gram-positive bacteria, including MRSA, were evaluated in three studies of patients with skin infections.^24–26^ Ceftaroline was compared with the combination of vancomycin and aztreonam in a pooled analysis of two identical RCTs in 1378 patients with complicated SSTI requiring admission to hospital (CANVAS 1 and 2).^24^ The trials were designed to demonstrate non-inferiority of ceftaroline (600 mg 12 hourly) compared with vancomycin (1 g 12 hourly adjusted according to local guidelines) given for 5–14 days. The primary outcome measure was clinical cure at a follow up visit 8–15 days after the last dose of antibiotics. Clinical cure rates in the pooled analysis met the predefined criteria for non-inferiority (lower limit of the 95% CI above −10%) and were 595/693 (85.9%) in the ceftaroline arm and 586/685 (85.5%) in the vancomycin/aztreonam arm (difference 0.3%; 95% CI −3.4% to 4.0%). Clinical cure was similar in 330 patients infected with MRSA [155/179 (86.6%) versus 124/151 (82.1%) in the ceftaroline and vancomycin/aztreonam modified intention-to-treat groups, respectively]. However, the small number of patients with MRSA in each group (fewer than 200), which resulted in a moderate risk of bias, precludes drawing reliable conclusions.

In an RCT recruiting 784 patients powered to demonstrate non-inferiority, ceftobiprole (500 mg 12 hourly) was compared with vancomycin (1 g 12 hourly adjusted according to local guidelines) in the treatment of complicated SSTI caused by Gram-positive organisms, including MRSA.^25^ The antibiotics were administered for 7–14 days and the primary outcome measure was clinical cure at an assessment visit performed 10–14 days after the end of treatment. Overall, the clinical cure rate was 263/282 (93.3%) in the ceftobiprole arm and 259/277 (93.5%) in the vancomycin arm (difference −0.2%; 95% CI −4.4% to +3.9%), which met the criteria for non-inferiority. There was no statistically significant difference in outcome between the two groups in patients infected with MRSA [cure rates of 56/61 (91.8%) in the ceftobiprole arm versus 54/60 (90%) in the vancomycin arm (difference 1.8%; 95% CI −8.4% to +12.1%)]. There is a moderate risk of bias due to the small number of patients with MRSA in each treatment arm, thereby undermining the reliability of the findings.

In a second RCT, ceftobiprole (500 mg 8 hourly) was compared with a combination of vancomycin (1 g 12 hourly adjusted according to local guidelines) plus ceftazidime (1 g 8 hourly) in the treatment of adults with a range of different complicated skin and soft tissue infections.^26^ Treatment was given for 7–14 days and the primary outcome was clinical cure assessed 7–14 days after the end of therapy. There were 547 patients in the ceftobiprole arm and 281 patients in the comparator arm. Of these, 87 and 36 patients respectively had infection due to MRSA. Overall clinical cure rates in the ITT population were similar for ceftobiprole (448/547 (81.9%) and vancomycin plus ceftazidime (227/281 (80.8%); difference 1.1%; 95% CI −4.5% to +6.7%). In the patients with MRSA, clinical cure rates were similar and were 78/87 (89.7%) in the ceftobiprole arm and 31/36 (86.1%) in the comparator arm (difference 3.6%; 95% CI −8.0% to +19.7%). Owing to the small number of patients in each group, there was a moderate-to-high risk of bias, which precludes drawing meaningful conclusions.

Dalbavancin, a new lipoglycopeptide, was studied in two identical non-inferiority trials comprising 1312 patients which were performed for licensing purposes (DISCOVER 1 and DISCOVER 2) and the results were pooled.^27^ Patients with SSTI requiring intravenous antibiotic therapy were given either dalbavancin 1 g on day 1 followed by 500 mg on day 8, or vancomycin 15 mg/kg 12 hourly for at least 3 days with an option to switch to oral linezolid 600 mg twice daily to complete 10–14 days of therapy. The primary endpoint was early clinical response as measured at 48–72 h. Overall, dalbavancin was not inferior to vancomycin/linezolid with very similar outcomes in the two treatment arms [cure rates of 525/659 patients (79.7%) treated with dalbavancin compared with 521/653 patients (79.8%) treated with vancomycin ± linezolid (difference −0.1%; 95% CI −4.5% to +4.2%)]. In the secondary analysis of the subset of patients with proven MRSA infection, cure rates assessed at the end of treatment were similar [72/74 patients (97.3%) in the dalbavancin group versus 49/50 patients (98%) in the vancomycin ± linezolid group]. Given that there were fewer than 100 patients in each arm, there would be a moderate risk of bias, thereby undermining the reliability of the findings.

Two almost identical large international multicentre RCTs powered to demonstrate non-inferiority compared a single dose of oritavancin (1200 mg) with vancomycin (1 g twice daily) given for 7–10 days in the treatment of patients with SSTIs (SOLO-I and SOLO-II, recruiting 968 and 1019 patients, respectively).^29^^,^^30^ In both studies oritavancin was demonstrated to be non-inferior to vancomycin with equivalent clinical response rates in both arms. In the SOLO-I study, the early clinical response in the modified intention-to-treat population was 391/475 (82.3%) for oritavancin and 378/479 (78.9%) for vancomycin (difference 3.4%; 95% CI −1.6% to +8.4%) and in SOLO-II the corresponding response rates were 403/503 (80.1%) for oritavancin and 416/502 (82.9%) for vancomycin (difference −2.7%; 95% CI −7.5% to +2.0%). In a pooled analysis, for those patients with proven infection caused by MRSA, the early clinical response at 48–72 h was 166/204 (81.4%) in the oritavancin arm versus 162/201 (80.6%) in the vancomycin arm (difference 0.8%; 95% CI −6.9% to +8.4%), which met the predefined criteria for non-inferiority.^28^

Two large international, multicentre studies powered to demonstrate non-inferiority and comprising 1867 adults with complicated SSTI requiring intravenous therapy compared telavancin (10 mg/kg/day) with vancomycin (1 g 12 hourly and then adjusted according to therapeutic drug monitoring) (the ATLAS studies); the results were pooled.^31^ Antibiotics were given for 10–14 days and the primary endpoint was cure as assessed 10–14 days after the completion of treatment. In the pooled analysis, telavancin met the criteria for non-inferiority; cure was reported in 658/745 (88.3%) clinically evaluable patients in the telavancin arm and 648/744 (87.1%) in the vancomycin arm (difference 1.2%; 95% −2.1 to +4.6%). Among patients with infection caused by MRSA, telavancin was non-inferior to vancomycin [cure rate of 252/278 patients (90.6%) in the telavancin group versus 260/301 patients (86.4%) in the vancomycin group (difference 4.1%; 95% CI −1.1% to +9.3%)].

Tedizolid was compared with linezolid in two studies powered to demonstrate non-inferiority in patients with SSTI caused by Gram-positive bacteria (ESTABLISH-1 and ESTABLISH-2).^32^^,^^33^ The primary outcome measure was early clinical response as assessed at 48–72 h according to FDA guidance. ESTABLISH-1 (*n = *667 patients) compared oral tedizolid (200 mg once daily for 6 days) with oral linezolid (600 mg twice daily for 10 days).^33^ ESTABLISH-2 (*n = *666 patients) was similar in terms of dosage and duration of therapy, but compared tedizolid with linezolid both given intravenously (with the option to de-escalate to oral therapy after administration of a minimum of two intravenous doses).^32^ In both of the studies the pre-defined non-inferiority criterion was a lower 95% CI of higher than −10% and demonstrated that tedizolid was non-inferior to linezolid. In ESTABLISH-1 the early clinical response was 259/332 (78%) for tedizolid and 255/335 (76.1%) for linezolid (difference 1.9%; 95% CI −4.5% to +8.3%) and in ESTABLISH-2 was 283/332 (85%) for tedizolid and 276/334 (83%) for linezolid (difference 2.6%; 95% CI −3.0% to +8.2%). For patients with infection caused by MRSA assessed at a follow up visit 7–10 days after the end of treatment, response rates in ESTABLISH-1 were 75/88 (85.2%) versus 77/90 (85.6%) in the tedizolid and linezolid arms, respectively (*P* value not reported) and, in ESTABLISH-2, 44/53 (83%) versus 44/56 (79%) in the tedizolid and linezolid arms, respectively (difference 4.4%; 95% CI −10.8% to +19.5%). Given that there were fewer than 100 patients in each arm, there would be a moderate risk of bias, thereby undermining the reliability of the findings.

Pleuromutilin antibiotics (lefamulin and retapamulin) were assessed in two RCTs.^15^^,^^34^ In the first trial,^34^ patients with ABSSSI were randomly assigned to receive either lefamulin 100 mg, or lefamulin 150 mg, or vancomycin (dosage adjusted according to standard practice in individual institutions) for 5–14 days. The primary outcome measure was clinical cure as assessed 10–14 days after the end of treatment. Overall cure rates were 54/60 (90%) in the lefamulin (100 mg) arm, 48/54 (88.9%) in the lefamulin (150 mg) arm and 47/51 (92.2%) in the vancomycin arm. MRSA was identified in 105/210 patients recruited. Cure rates in the MRSA subgroup were similar in all of the study arms [lefamulin (100 mg) 29/34 (85.3%), lefamulin (150 mg) 28/32 (87.5%) and vancomycin 32/39 (82.1%)]; a *P* value was not reported. However, there is a high risk of bias due to the small number of patients with MRSA in each treatment arm, thereby undermining the reliability of the findings. At the time of writing, lefamulin is awaiting regulatory approval.

The second trial^15^ is discussed in Section 4.1.1 in relation to impetigo. Topical retapamulin 1% administered twice daily for 5 days was shown to be inferior to oral treatment with linezolid 600 mg 12 hourly for 10 days (or equivalent dose adjusted for age in children) in an RCT including adults and children with either infected wounds or impetigo caused by MRSA.^15^ The primary outcome measure was clinical cure at follow up 7–9 days after the completion of therapy; 410 patients were enrolled and 285 of these had infected wounds. Across the study population infected with MRSA, the clinical cure rate in the retapamulin group [41/72 (56.9%); ITT MRSA] was significantly lower than that in the oral linezolid group [32/38 (84.2%); difference −27.3%; 95% CI −45.8% to −8.7%] (there was no differentiation between infected wounds and impetigo). There is a moderate-to-high risk of bias due to the small number of patients with MRSA in each treatment arm, thereby undermining the reliability of the findings.

Delafloxacin was assessed in three studies included in the current systematic review.^35–37^ In the first, delafloxacin 300 mg was shown to be non-inferior to vancomycin 15 mg/kg plus aztreonam 2 g (each given 12 hourly for 5–14 days) in a RCT comprising 660 patients with ABSSSI.^35^ The primary outcome measure was clinical response at 48–72 h in the intention-to-treat population, and the predefined criterion for non-inferiority was that the lower 95% CI of the difference between the treatment arms was greater than −10%. Clinical response was seen in 259/331 (78.2%) patients in the delafloxacin arm and 266/329 (80.9%) patients in the vancomycin plus aztreonam arm (difference −2.6%; 95% CI −8.8% to +3.6%). In the subgroup of patients with proven MRSA infection, clinical response was seen in 190/220 (86.4%) patients in the delafloxacin arm and in 199/225 (88.4%) patients in the vancomycin plus aztreonam arm (difference −2.0%; 95% CI −8.39% to +4.16%).

In the second RCT, two different dosing regimens of delafloxacin were compared with tigecycline in the treatment of a variety of different complicated skin and soft tissue infections in a study performed in 2008 and published in 2015.^37^ Patients were randomized 1 : 1 : 1 to receive intravenous delafloxacin 300 mg 12 hourly, or delafloxacin 450 mg 12 hourly, or tigecycline 100 mg initially followed by 50 mg 12 hourly. Treatment was continued for 5–14 days and the primary outcome was clinical cure as assessed at a visit 7–14 days after completion of therapy. Cure rates in the clinically evaluable population were 33/35 (94.3%) in the delafloxacin 300 mg arm, 37/40 (92.5%) in the delafloxacin 450 mg arm and 31/34 (91.2%) in the tigecycline arm (*P > *0.5 by Fisher’s exact test). In the patients with infection due to MRSA, clinical cure was seen in 13/14 (92.9%), 19/20 (95.0%) and 12/14 (85.7% in the three arms respectively (*P > *0.5). There is a high risk of bias due to the small number of patients with MRSA in each treatment arm, thereby undermining the reliability of the findings.

In the third RCT, patients with acute bacterial skin and skin structure infections were randomized (1 : 1 : 1) to intravenous delafloxacin 300 mg, or linezolid 600 mg, or vancomycin 15 mg/kg (actual body weight) administered 12 hourly for 5–14 days.^36^ Vancomycin doses were adjusted to achieve a trough serum concentration of 15-20 mg/L. The primary outcome was clinical response as assessed at a visit following the completion of treatment. Overall, clinical response was seen in 57/81 (70.4%) patients treated with delafloxacin, 50/77 (64.9%) patients treated with linezolid and 53/98 (54.1%) patients treated with vancomycin. The response with delafloxacin was assessed as significantly higher than with vancomycin (*P < *0.05 by the Cochran–Mantel–Haenszel test). However, this difference was not observed in the subset of patients with infection caused by MRSA. Response rates in these patients were 19/29 (65.5%), 21/34 (61.8%) and 21/32 (65.6%) in the three groups, respectively. There is a high risk of bias due to the small number of patients with MRSA in each treatment arm, thereby undermining the reliability of the findings.

Iclaprim, a novel diaminopyridimine antibiotic that inhibits dihydrofolate reductase, has been studied in two similar licensing RCTs in patients with ABSSSI (REVIVE-1 and REVIVE-2).^38^^,^^39^ Iclaprim given intravenously at a dose of 80 mg twice daily was compared with vancomycin 15 mg/kg twice daily (dose adjusted according to therapeutic drug monitoring), each given for 5–14 days. The primary endpoint was early clinical response at 48–72 h in the intention-to-treat population and the studies were powered to demonstrate non-inferiority. In the two trials, a total of 1198 patients were recruited and 272 had infection caused by MRSA. Iclaprim was non-inferior to vancomycin in the total patient population [clinical cure in the REVIVE-1 study was 241/298 (80.9%) in the iclaprim arm and 243/300 (81%) in the vancomycin arm (difference −0.1%; 95% CI −6.42% to +6.17%), while in the REVIVE-2 study, clinical cure was 231/295 (78.3%) in the iclaprim arm and 234/305 (76.7%) in the vancomycin arm (difference 1.58%; 95% CI −5.1% to +8.26%)]. Within the subgroup with MRSA, clinical cure in the REVIVE-1 study was 59/73 (80.8%) in the iclaprim arm and 50/61 (82%) in the vancomycin arm [(difference −1.15%; 95% CI −17.9% to +15.8%)], while in the REVIVE-2 study, clinical cure was 61/69 (88.4%) iclaprim arm and 53/69 (76.8%) in the vancomycin arm [difference 11.6%; 95% CI −5.8% to +28.5%)]. Given that there were fewer than 100 patients in each arm, there would be a moderate risk of bias, thereby undermining the reliability of the findings.

At the time of writing, iclaprim had not been licensed for clinical use; further safety data on hepatic toxicity are also awaited.

##### Quality of the evidence

Large RCTs evaluating different treatment options for MRSA causing SSTI were the most frequent trials identified; however, these were designed to determine non-inferiority and the level of evidence was variable.

Trials assessing the clinical efficacy of ceftaroline, delafloxacin, oritavancin, and telavancin had an adequate number of participants with MRSA in each arm (quality of evidence: 1+). The reliability of findings from trials of other agents were undermined by a moderate-to-high risk of bias due to a smaller number of patients with MRSA in each treatment arm (quality of evidence: 1−).

##### Recommendations

- For severe cellulitis/soft tissue infection caused by MRSA use intravenous glycopeptides (vancomycin or teicoplanin) (strong recommendation).
- Use linezolid (oral or intravenous) or daptomycin (intravenous) as an alternative (strong recommendation).
- Consider tigecycline as an alternative when first- and second-line agents are contraindicated, and the isolate is susceptible (weak recommendation).
- Consider clindamycin, co-trimoxazole, or doxycycline as oral agents (when the isolate is susceptible) for treatment of patients with mild skin and soft tissue infection caused by MRSA, or for oral stepdown therapy (weak recommendation).
- Consider recently licensed agents such as ceftaroline, delafloxacin, oritavancin, or telavancin as alternative options for treatment of cellulitis/soft tissue infection caused by MRSA (weak recommendation).
- No recommendations can be made on the use of ceftobiprole, dalbavancin and tedizolid over standard therapeutic agents in the treatment of SSTI caused by MRSA.

### 4.2 Urinary tract infections

####  

##### Evidence

There is a lack of evidence on the management of MRSA UTIs. No new evidence was identified for the treatment of such infection, but the previous guidelines were reviewed. The guideline development group feel that the detection of MRSA in the urine should lead to an investigation of the cause. MRSA might be shed in the urine as a result of a bacteraemia or the presence of the bacterium in the urine may simply represent colonization, particularly in patients with long-term catheters. MRSA bacteriuria may also be secondary to an anatomic abnormality of the urinary tract. While linezolid may occasionally be used for treatment of UTI caused by vancomycin-resistant enterococci, we advise caution in the use of this agent to treat UTI caused by MRSA due to low levels of excretion by the kidneys.

##### Quality of the evidence

UTI caused by MRSA is not well represented in clinical trials (quality of evidence: 4).

##### Recommendations

- Exclude the presence of MRSA bacteraemia before commencing treatment of MRSA isolated from urine (weak recommendation).
- Consider treating a genuine lower UTI caused by MRSA with an oral agent, such as doxycycline, trimethoprim, ciprofloxacin, or co-trimoxazole, according to susc eptibility (weak recommendation).
- For complicated UTI caused by MRSA consider intravenous glycopeptides (vancomycin or teicoplanin) as the first-line treatment (weak recommendation).
- When a glycopeptide is contraindicated consider daptomycin as an alternative agent if intravenous therapy is required (weak recommendation).
- Linezolid is not recommended for the treatment of MRSA UTI, given its poor excretion by the kidney (weak recommendation).
- For catheter-associated UTI caused by MRSA, whenever possible/practicable replace the catheter, with or without a single dose of gentamicin if the MRSA isolate is known to be susceptible (weak recommendation). Consider a single dose of glycopeptide (vancomycin or teicoplanin) as an alternative if the isolate is resistant to gentamicin or there are other contraindications (weak recommendation).

### 4.3 Bone and joint infections

####  

##### Evidence

Bone and joint infections caused by MRSA can be difficult to treat and patients may require prolonged courses of antimicrobial therapy. Our recommendations do not include the management of the specialist conditions such as prosthetic joint infection and diabetic foot infection.

Paul *et al.* (2015)^40^ performed a RCT of co-trimoxazole compared with vancomycin in patients with a range of severe infections caused by MRSA. Co-trimoxazole was initially administered intravenously at a dosage of 1920 mg 12 hourly and this was converted to an oral formulation at the same dosage at a time chosen by the treating physician. The vancomycin dosing was 1 g 12 hourly intravenously with target pre-dose serum concentrations of 10-20 mg/L; the duration of therapy was not reported. The trial was powered for non-inferiority across a range of infections and therefore not powered to determine utility of co-trimoxazole in bone or joint infection alone. This trial included 39 and 32 patients with MRSA bone and joint infection, randomized to co-trimoxazole or vancomycin, respectively (unpublished data cited in Paul *et al.*^40^). The primary outcome measure was clinical treatment failure at 7 days, and this occurred in 11/39 (28%, co-trimoxazole) and 7/32 (22%, vancomycin) patients, respectively. A secondary outcome of mortality at 30 days did not differ between groups [2/39 (5%) for co-trimoxazole and 1/32 (3%) for vancomycin]. The differences were not statistically significant, but in any event, the study was associated with a high risk of bias, owing to the low numbers of patients, thereby precluding us from drawing any meaningful conclusions.

Owing to its biofilm penetration rifampicin has been recommended in previous UK guidelines^1^ as adjunctive therapy in patient with MRSA bone or joint infections, particularly where metalwork is implanted. No evidence fulfilling the inclusion criteria on the use of rifampicin to treat bone infection was identified during the current systematic review.

There is increasing experience in the UK of the use of dalbavancin to treat bone infection. Due to its long half-life and suitability for weekly administration, it is used when other drugs cannot be easily administered. No evidence fulfilling the inclusion criteria on the use of dalbavancin to treat bone infection was identified during the current systematic review.

##### Quality of the evidence

Recent clinical trials have not been sufficiently powered to provide new evidence for the treatment of bone and joint infections caused by MRSA (quality of evidence: 4).

##### Recommendations

- Use a multidisciplinary approach for treatment of MRSA bone and joint infections, including surgery or drainage where indicated (strong recommendation).
- For bone and joint infections caused by MRSA use intravenous glycopeptides (vancomycin or teicoplanin) as the first-line choice of treatment (strong recommendation).
- Consider 2 weeks of intravenous glycopeptide (vancomycin or teicoplanin), followed by further intravenous or oral antibiotics to complete a total treatment course of a minimum of 4 weeks for septic arthritis or 6 weeks for osteomyelitis (weak recommendation).
- Use therapeutic drug monitoring to ensure that non-toxic, therapeutic pre-dose serum concentrations of 15–20 mg/L for vancomycin, or 20–40 mg/L for teicoplanin are achieved (strong recommendation).
- When a glycopeptide is contraindicated consider daptomycin or linezolid as alternative agents (weak recommendation).
- Use clindamycin, co-trimoxazole, doxycycline, or linezolid as oral options to complete treatment when the MRSA isolate is known to be susceptible (strong recommendation).
- Do not use rifampicin, fusidic acid or a quinolone as a single oral agent; use in combination with other agents to which the isolate is susceptible (strong recommendation).

### 4.4 Bacteraemia

####  

##### Evidence

Four studies met our inclusion criteria in this clinical setting.^40–43^ Paul *et al.* (2015)^40^ enrolled 91 patients with MRSA bacteraemia in an RCT. Fifty patients were randomized to vancomycin (target pre-dose serum concentrations 10–20 mg/L) and 41 to co-trimoxazole (1920 mg 12 hourly intravenously initially then oral or intravenously). In the intention-to-treat analysis, 23/41 (56%) and 20/50 (40%) patients in the vancomycin and co-trimoxazole groups respectively experienced clinical treatment failure at day 7 [effect estimate 1.4 (95% CI 0.9–2.16). The secondary outcome of all-cause mortality at 30 days was non-significantly higher in the co-trimoxazole group [14/41 (34%) versus 9/50 (18%), effect estimate 1.9 (95% CI 0.9–3.9)], leading the study authors to advise that this antibiotic is not used alone to treat patient with MRSA bacteraemia. The small numbers of patients per study arm and associated high risk of bias invalidates the statistical analysis and confounds the impact of this recommendation. HERE

Thwaites *et al.* (2018)^43^ allocated patients with *S. aureus* bacteraemia to a standard first-line regimen together with adjunctive therapy comprising either rifampicin or placebo. Of the 47 patients with infections caused by MRSA, 26 received rifampicin and 21 received placebo. Nine of 26 (35%) patients who were given adjunctive rifampicin and 3/21 (14%) prescribed adjunctive placebo met the primary outcome measure at 12 weeks (clinically defined treatment failure or disease recurrence, or death) (*P > *0.05). The authors concluded that the addition of adjunctive rifampicin to standard therapy did not improve clinical outcomes in patients with *S. aureus* bacteraemia (SAB), but conclusions cannot be presumed to extrapolate to MRSA. The low numbers of patients and associated high risk of bias preclude drawing any meaningful conclusions regarding the intervention of adjunctive rifampicin in patients with MRSA bacteraemia.

*In vitro* and animal studies have suggested the potential for synergy when vancomycin is combined with β-lactam antibiotics for the treatment of patients with MRSA bacteraemia.^44^^,^^45^ Davis *et al.* (2016)^41^ report the results of a pilot RCT (CAMERA) designed to investigate the effect of an adjunctive β-lactam in this setting. Three hundred and eighty patients were screened and 60 were recruited. All participants received intravenous vancomycin 1.5 g 12 hourly, with 29 patients receiving adjunctive placebo (control arm) and 31 patients receiving adjunctive flucloxacillin 2 g 6 hourly (combination arm), for 7 days. The duration of bacteraemia was 3 days in the control arm and 1.94 days in the combination arm in the intention-to-treat analysis (*P = *0.06). As the high risk of bias arising from enrolment of small numbers of patients precludes drawing any meaningful conclusions and as the results were inconclusive.

Rehm *et al.* (2008)^42^ compared daptomycin (6 mg/kg/day) with vancomycin combined with low-dose gentamicin in patients with bacteraemia or endocarditis due to MRSA. In this RCT, 32 patients with MRSA bacteraemia received daptomycin and were compared with 33 patients with MRSA bacteraemia who received the usual therapy of vancomycin combined with low-dose gentamicin. Six weeks following the conclusion of therapy, clinical success was recorded 16/32 (50%) in the daptomycin arm and 11/33 (33%) in the vancomycin/gentamicin arm. In this trial the results were separated into complicated and uncomplicated bacteraemia, but the small numbers perhaps preclude sub-analysis. Expert Infectious Diseases Society of America (IDSA) opinion suggests a higher dose of daptomycin of 8–10 mg/kg/day may be required, particularly in complicated bacteraemia.^16^ The small overall numbers of patients per study arm and associated high risk of bias invalidates the statistical analysis and no recommendation is based on this trial result.

The evidence regarding teicoplanin treatment of MRSA bacteraemia is limited. There is no robust evidence of inferiority of teicoplanin compared with vancomycin.

Evidence to support course duration for treatment of MRSA bacteraemia is limited, although it is generally agreed that a longer treatment duration is needed for complicated bacteraemia. For definitions of uncomplicated and complicated bacteraemia, the reader is referred to the clinical practice guidelines by the IDSA for the treatment of MRSA infections in adults and children.^16^

##### Quality of the evidence

Recent clinical trials have not been sufficiently powered to provide new evidence for the treatment of bacteraemia caused by MRSA and optimum duration of therapy remains subjective (quality of evidence: 4).

Treatment of MRSA bacteraemia with co-trimoxazole may be associated with a higher rate of clinical failure compared with vancomycin; however, this association has yet to be confirmed (quality of evidence: 1−).

##### Recommendations

- Use intravenous vancomycin for uncomplicated bacteraemia caused by MRSA (strong recommendation).
- When vancomycin is contraindicated use linezolid as an alternative first-line choice of treatment (strong recommendation).
- When first-line agents are contraindicated consider daptomycin or teicoplanin (weak recommendation).
- Do not use co-trimoxazole alone as a first-line agent for MRSA bacteraemia, however, consider using it as an oral step-down when the MRSA isolate is known to be susceptible (weak recommendation).
- Consider a minimum duration of 14 days of antibiotic therapy for uncomplicated bacteraemia and a minimum duration of 28 days for complicated bacteraemia caused by MRSA (weak recommendation).

### 4.5 Infective endocarditis

####  

##### Evidence

Current BSAC endocarditis guidelines^46^ advise the use of vancomycin for vancomycin-susceptible native or prosthetic valve MRSA endocarditis. If the patient cannot tolerate vancomycin or if the isolate is not vancomycin susceptible then daptomycin is recommended as an alternative in combination with a second agent chosen according to antibiotic susceptibility testing. The guidelines recommend a minimum duration of 4 weeks for patients with native valve endocarditis and 6 weeks for those with prosthetic valve endocarditis.

##### Recommendation

The reader is referred to the most recent version of the BSAC endocarditis guidelines.

### 4.6 Respiratory tract infections

#### 4.6.1 Necrotizing pneumonia

##### Evidence

MRSA necrotizing pneumonia (e.g. in association with Panton–Valentine leucocidin) is a life-threatening disease that requires urgent treatment. No new evidence that allows existing guidelines to be updated/modified has been identified in the current systematic review. The 2008 UK MRSA treatment guidelines^1^ recommended the use of vancomycin or linezolid, but the authors expressed concerns regarding the efficacy of both drugs. Previous Public Health England (PHE) guidelines, which advised a combination of clindamycin, linezolid and rifampicin, have not been updated since 2008 and we do not recommend that these are used to guide treatment.

##### Quality of the evidence

Treatment of necrotizing pneumonia caused by MRSA is not well represented in clinical trials (quality of evidence: 4).

##### Recommendations

- For necrotizing pneumonia caused by MRSA, use intravenous vancomycin or linezolid (strong recommendation).
- Consider addition of a toxin-inhibiting agent, such as clindamycin or rifampicin when the MRSA isolate is known to be susceptible (weak recommendation).

#### 4.6.2 Nosocomial pneumonia

##### Evidence

Wunderink *et al.* (2012)^47^ performed a large RCT that enrolled 1184 patients with all-cause nosocomial pneumonia in the initial recruitment. Patients were randomized to receive either intravenous linezolid (600 mg 12 hourly) or intravenous vancomycin (15 mg/kg 12 hourly) for 7–14 days (or 21 days in the case of associated bacteraemia). The primary outcome measure for end-of-study clinical success was analysed according to those patients later identified as having confirmed MRSA (a proportion of whom had MRSA combined with other pathogens). This included 348 patients (linezolid, *n = *172; vancomycin, *n = *176). In the per protocol population, 95/165 (58%) in the linezolid arm and 81/174 (46.5%) in the vancomycin arm achieved clinical success at end of study (*P = *0.042). Mortality at 60 days did not differ between the modified intention-to-treat and the intention-to-treat groups. Moreover, the small number of patients with MRSA in each group (fewer than 200), which resulted in a moderate risk of bias, precludes drawing reliable conclusions.

Rubinstein *et al*. (2011)^48^ randomized 1532 patients with nosocomial pneumonia, of whom 290 had evidence of MRSA infection, to intravenous telavancin 10 mg/kg/day (*n = *136) or vancomycin 1 g 12 hourly (*n = *154) for 7–21 days. In the patients with MRSA infection, 104/136 (76.5%) treated with telavancin and 115/154 (74.7%) treated with vancomycin were assessed as cured at the test of cure/follow up visit. Although these numbers are larger than those in other studies in this clinical setting, they are not adequately powered to demonstrate non-inferiority of telavancin to vancomycin in patients with MRSA pneumonia; indeed, the number of patients in each group (fewer than 200) resulted in a moderate risk of bias, which precludes drawing reliable conclusions. Overall, there was an increase in serious adverse events (septic shock, respiratory failure or multiorgan failure) and treatment-emergent adverse events (diarrhoea, anaemia, hypokalaemia, constipation, or renal impairment) in patients receiving telavancin (234/751; 31%) compared with those receiving vancomycin (197/752; 26%) in this trial (no *P* value available).

Awad *et al.* (2014)^49^ completed an RCT to compare ceftobiprole with ceftazidime plus linezolid in hospital-acquired pneumonia (HAP). Infection was caused by MRSA in 41 of 391 patients in the ceftobiprole group and in 48 of 390 patients in the ceftazidime/linezolid group (although MRSA may not have been the only pathogen in patients in either group); of these 89 patients with HAP, 29 had ventilator-associated pneumonia (VAP). In the subset of 55 patients for whom there was a microbiologically evaluable outcome: 13/27 (48%) had microbiological eradication and 17/27 (63%) had clinical cure with ceftobiprole; 16/28 (57%) had microbiological eradication and 18/28 (64%) had clinical cure with ceftazidime plus linezolid. The report quotes a subset analysis for early clinical improvement at day 4 in the clinically evaluable patients with HAP (without VAP) who had MRSA and found a statistically significant difference in outcome in patients treated with ceftobiprole (18/19; 95%) versus those treated with ceftazidime plus linezolid (10/19; 53%) (difference 42%, 95% CI 17.5%–66.7%). However, this difference was not found in the intention-to-treat population of those with MRSA (improvement in 22/28 (78%) versus 19/32 (59%) with ceftobiprole or ceftazidime plus linezolid respectively, difference 19%, 95% CI −3.6% to 42%). There is a high risk of bias with these small numbers. No recommendations are based on the results from this trial.

Daptomycin is not licensed for treatment of respiratory infections due to inhibitory interaction of the molecule with lung surfactant.^50^

##### Quality of the evidence

Nosocomial pneumonia caused by MRSA treated with linezolid is associated with a significantly higher clinical response rate compared with nosocomial pneumonia treated with vancomycin, although this observation is based on only one RCT for which the risk of bias, based on the number of patients with MRSA enrolled into each group, is moderate (quality of evidence: 1−).

##### Recommendations

- In the absence of at least one additional RCT confirming the superiority of linezolid over vancomycin for nosocomial pneumonia caused by MRSA, ideally associated with a low risk of bias, we have opted to recommend either intravenous vancomycin or linezolid as first-line therapy (weak recommendation).
- Do not use daptomycin to treat nosocomial pneumonia caused by MRSA, as it is inactivated by lung surfactant (strong recommendation).
- No recommendations can be made on the use of ceftobiprole over standard therapeutic agents in the treatment of HAP caused by MRSA.

#### 4.6.3 Ear, nose and throat or upper respiratory tract infections

##### Evidence

MRSA-associated ear, nose and throat or upper respiratory tract infections are rare, although they may be complicated by skull penetration or brain abscess formation. No new evidence that might inform our recommendations was identified in the current systematic review.

##### Quality of the evidence

Treatment of ear, nose and throat or upper respiratory tract infections caused by MRSA is not well represented in clinical trials (quality of evidence: 4).

##### Recommendations

- For severe MRSA-associated ear, nose and throat or upper respiratory tract infections consider intravenous glycopeptide (vancomycin or teicoplanin) or linezolid (weak recommendation).
- For minor/less severe infections consider co-trimoxazole or doxycycline as an oral option when the MRSA isolate is known to be susceptible (weak recommendation).

#### 4.7 Central nervous system and eye disease

##### 4.7.1 Intracranial or spinal infections

###### Evidence

Infections in this category include brain abscess, subdural empyema, spinal epidural abscess and vertebral osteomyelitis. No new evidence which might inform our recommendations for the treatment of patients with MRSA infection in these clinical settings was identified in the current systematic review.

Whenever clinically possible, source control is necessary for intracranial and spinal infections.

###### Quality of the evidence

Treatment of intracranial and spinal infections caused by MRSA are not well represented in clinical trials (quality of evidence: 4).

###### Recommendations

- Whenever clinically possible, source control is necessary for intracranial and spinal infections (strong recommendation).
- Unless surgical intervention is contraindicated use incision and drainage for treatment of intracranial and spinal infections caused by MRSA (strong recommendation).
- In the absence of neurological deficits consider treating small epidural abscesses with antibiotics alone (weak recommendation).
- For treatment of intracranial and spinal infections caused by MRSA consider intravenous vancomycin or linezolid as the first-line choice of treatment (weak recommendation).

##### 4.7.2 Meningitis

###### Evidence

No new evidence that might inform our recommendations for the treatment of patients with meningitis caused by MRSA was identified in the current systematic review.

Shunt infection was not considered in this review. Oritavancin has demonstrated efficacy in animals and may have a role to play in this clinical setting, but that role is not currently clear.

###### Quality of the evidence

Treatment of meningitis caused by MRSA is not well represented in clinical trials (quality of evidence: 4).

###### Recommendations

- For meningitis caused by MRSA use intravenous vancomycin (strong recommendation). For severe infection, consider adding rifampicin according to susceptibility (weak recommendation).
- Use therapeutic drug monitoring to ensure that non-toxic, therapeutic pre-dose serum concentrations (15-20 mg/L) of vancomycin are achieved (strong recommendation).
- In severe cases or when the patient fails to respond to intravenous vancomycin, transfer the patient to a neurosurgical centre for instillation of vancomycin directly into the ventricles (strong recommendation).
- Do not use clindamycin, chloramphenicol or linezolid to treat meningitis caused by MRSA (strong recommendation). These drugs are not bactericidal, such activity being a requirement of antibiotics used as therapy of patients with meningitis.
- No recommendation can be made for the use of teicoplanin in this clinical setting.

##### 4.7.3 Eye disease

###### Evidence

MRSA eye disease is rare. In the event, no new evidence which might inform our recommendations for the treatment of patients with eye infection caused by MRSA was identified in the current systematic review.

Endophthalmitis can represent dissemination secondary to bacteraemia and this should always be considered when a patient is diagnosed with this disease.

###### Quality of the evidence

Treatment of eye disease caused by MRSA is not well represented in clinical trials (quality of evidence: 4).

###### Recommendations

- For superficial MRSA eye disease consider gentamicin or chloramphenicol eye drops according to isolate susceptibility (weak recommendation).
- For deep-seated eye infections caused by MRSA consider a multidisciplinary approach comprising specialist ophthalmologists and infection specialists (weak recommendation).
- For deep-seated eye infections caused by MRSA consider intravitreal vancomycin and systemic quinolones according to susceptibility (weak recommendation).
- Consider oral linezolid as a treatment option, recognizing that there is limited evidence of efficacy in MRSA infection at this site (weak recommendation).

### 5. Implementation of these guidelines

#### 5.1 How can the guidelines be used to improve clinical effectiveness?

These guidelines can be used to inform antibiotic treatment policies and provide standards for clinical audit. Areas of additional research are identified, thereby directing future research necessary for the provision of high-quality, evidence-based recommendations.

#### 5.2 How much will implementation of the guidelines cost?

Implementation of recommendations in the updated guideline is not anticipated to be associated with any additional costs compared with the previous guideline; however, treatment using newer anti-MRSA agents may be associated with higher costs than established MRSA treatments.

#### 5.3 Summary of suggested audit measures

- Proportion of cases of impetigo caused by MRSA which were treated with topical antiseptic (Aim >80%).
- Proportion of cases of impetigo caused by MRSA which were treated with topical antibiotic (Aim <20%).
- Proportion of abscesses >5 cm in diameter caused by MRSA which underwent incision and drainage within 48 h of diagnosis (Aim >95%).
- Proportion of patients with abscesses that are drained or that are <5 cm in diameter treated with systemic antibiotics in the absence of a systemic response (fever and/or cellulitis) and/or immunodeficiency (Aim <20%).
- Proportion of patients with MRSA UTI who have a blood culture taken to exclude MRSA bacteraemia (Aim 100%).
- Proportion of patients with MRSA UTI who are treated with an agent which is effectively excreted in the urine (Aim 100%).
- Proportion of patients with MRSA bone and joint infection discussed at a multidisciplinary team (MDT) meeting (Aim >95%).

#### 5.4 E-Learning tools

An assessment tool is available to identify compliance with the recommendations within this guideline (available as Supplementary data at *JAC-AMR* online). Information collected by the tool informs service providers where their service is doing well, where improvements could be made, or where support is required.

### 6. Evidence gaps and further research

It is disappointing that efforts to produce robust, evidence-based recommendations have been limited by the absence of adequate numbers of well-designed and well-conducted clinical trials. Evidence gaps have been identified and further research is urgently needed in the following areas:

- The role of teicoplanin: to determine if there a tendency to under-dose, to identify the optimum dosage of teicoplanin, to support teicoplanin therapeutic drug monitoring and identify peak and trough values, to identify the circumstances when ‘actual body weight’ or ‘ideal body weight’ are used in dosage calculations.
- Gentamicin regimens in patients with renal impairment.
- The role of linezolid: to investigate an association between the duration of therapy with linezolid and adverse events, and the requirement for, and interpretation of, tests to monitor for adverse events.
- Management of MRSA in chronic wounds: to identify agents that are effective against biofilms, topical agents that are effective in treatment, and to determine the utility of topical fusidic acid in primary care and dermatology.
- Establish the difference between MRSA colonization and infection in the urine and the role of trimethoprim and ciprofloxacin in UTI management.
- To identify ways to differentiate between MRSA colonization and infection in the lung.
- The treatment of CNS infection in view of the limitations of the agents available and identification of optimal treatment in relation to CNS penetration.
- The use of older medicines, such as co-trimoxazole or chloramphenicol, in the management of MRSA infection.
- Duration of treatment: duration is unclear in several conditions, including when to switch from intravenous to oral agents and patient factors associated with such a switch.
- Agents most appropriate for administration within outpatient parenteral antimicrobial therapy (OPAT) for management of MRSA infection.
- Agents to use when there is evidence of allergy/hypersensitivity/intolerance to the first-line agents.
- Vancomycin-resistant *S. aureus.*

## 7. Conclusions

The incidence of MRSA has decreased considerably in the UK since the publication of the 2008 MRSA guidelines. Since 2008 there has been a change in clinical management of MRSA with linezolid and daptomycin available more widely. Several new antimicrobial agents with activity against MRSA have been licensed, but the evidence to support their routine use is limited. For reasons that are unclear, community strains of MRSA, such as PFGE strain type USA300, have remained uncommon in the UK. Evidence was found to support the use of antibiotic treatment in abscesses caused by USA300 and, should this become more common in the UK, it may then be necessary to recommend adjunctive antibiotics for the management of abscesses.

## Supplementary Material

dlaa114_Supplementary_Data

## References

[1] Gould FK , Brindle R, Chadwick PR et al Guidelines (2008) for the prophylaxis and treatment of methicillin-resistant *Staphylococcus aureus* (MRSA) infections in the United Kingdom. J Antimicrob Chemother 2009; 63: 849–61. 19282331 10.1093/jac/dkp065

[2] Duerden B , Fry C, Johnson AP et al The control of methicillin-resistant *Staphylococcus aureus* blood stream infections in England. Open Forum Infect Dis 2015; 2: ofv035. 26380336 10.1093/ofid/ofv035 PMC4567090

[3] Toleman MS , Reuter S, Coll F et al Systematic surveillance detects multiple silent introductions and household transmission of methicillin-resistant *Staphylococcus aureus* USA300 in the East of England. J Infect Dis 2016; 214: 447–53. 27122590 10.1093/infdis/jiw166 PMC4936647

[4] Cochrane. Cochrane Handbook for Systematic Reviews of Interventions version 6.0 (updated July 2019). 2019. www.training.cochrane.org/handbook.

[5] Higgins JP , Altman DG, Gotzsche PC et al The Cochrane Collaboration's tool for assessing risk of bias in randomised trials. Bmj 2011; 343: d5928. 22008217 10.1136/bmj.d5928 PMC3196245

[6] Gaskell H , Derry S, Wiffen PJ et al Single dose oral ketoprofen or dexketoprofen for acute postoperative pain in adults. Cochrane Database Syst Rev 2017; 5: CD007355. 28540716 10.1002/14651858.CD007355.pub3 PMC6481461

[7] Brouwers MC , Kho ME, Browman GP et al AGREE II: advancing guideline development, reporting and evaluation in health care. CMAJ 2010; 182: E839–42. 20603348 10.1503/cmaj.090449 PMC3001530

[8] SIGN. SIGN50: a guideline developer’s handbook. 2019. http://www.sign.ac.uk.

[9] Andrews J , Guyatt G, Oxman AD et al GRADE guidelines: 14. Going from evidence to recommendations: the significance and presentation of recommendations. J Clin Epidemiol 2013; 66: 719–25. 23312392 10.1016/j.jclinepi.2012.03.013

[10] Nathwani D , Morgan M, Masterton RG et al Guidelines for UK practice for the diagnosis and management of methicillin-resistant *Staphylococcus aureus* (MRSA) infections presenting in the community. J Antimicrob Chemother 2008; 61: 976–94. 18339633 10.1093/jac/dkn096

[11] FDA. Guidance for Industry. Acute Bacterial Skin and Skin Structure Infections: Developing Drugs for Treatment. 2013. https://www.fda.gov/downloads/Drugs/GuidanceComplianceRegulatoryInformation/Guidances/UCM071185.pdf.

[12] UK Governement. Management and treatment of common infections. Antibiotic guidance for primary care: for consultation and local adaptation. https://www.gov.uk/government/publications/managing-common-infections-guidance-for-primary-care.

[13] Koning S , van der Sande R, Verhagen AP et al Interventions for impetigo. Cochrane Database Syst Rev 2012; 1: CD003261. 22258953 10.1002/14651858.CD003261.pub3 PMC7025440

[14] Czekaj T , Ciszewski M, Szewczyk EM. *Staphylococcus haemolyticus* - an emerging threat in the twilight of the antibiotics age. Microbiology 2015; 161: 2061–8. 26363644 10.1099/mic.0.000178

[15] Tanus T , Scangarella-Oman NE, Dalessandro M et al A randomized, double-blind, comparative study to assess the safety and efficacy of topical retapamulin ointment 1% versus oral linezolid in the treatment of secondarily infected traumatic lesions and impetigo due to methicillin-resistant Staphylococcus aureus. Adv Skin Wound Care 2014; 27: 548–59. 25396674 10.1097/01.ASW.0000456631.20389.ae

[16] Stevens DL , Bisno AL, Chambers HF et al Practice guidelines for the diagnosis and management of skin and soft tissue infections: 2014 update by the Infectious Diseases Society of America. Clin Infect Dis 2014; 59: e10-52. 24973422 10.1093/cid/ciu444

[17] Chen AE , Carroll KC, Diener-West M et al Randomized controlled trial of cephalexin versus clindamycin for uncomplicated pediatric skin infections. Pediatrics 2011; 127: e573-80. 21339275 10.1542/peds.2010-2053 PMC3387913

[18] Daum RS , Miller LG, Immergluck L et al A placebo-controlled trial of antibiotics for smaller skin abscesses. N Engl J Med 2017; 376: 2545–55. 28657870 10.1056/NEJMoa1607033 PMC6886470

[19] Holmes L , Ma C, Qiao H et al Trimethoprim-sulfamethoxazole therapy reduces failure and recurrence in methicillin-resistant *Staphylococcus aureus* skin abscesses after surgical drainage. J Pediatr 2016; 169: 128–34.e1. 26578074 10.1016/j.jpeds.2015.10.044

[20] Talan DA , Moran GJ, Krishnadasan A et al Subgroup analysis of antibiotic treatment for skin abscesses. Ann Emerg Med 2018; 71: 21–30. 28987525 10.1016/j.annemergmed.2017.07.483 PMC5741525

[21] Talan DA , Mower WR, Krishnadasan A et al Trimethoprim-sulfamethoxazole versus placebo for uncomplicated skin abscess. N Engl J Med 2016; 374: 823–32. 26962903 10.1056/NEJMoa1507476 PMC4851110

[22] Itani KM , Dryden MS, Bhattacharyya H et al Efficacy and safety of linezolid versus vancomycin for the treatment of complicated skin and soft-tissue infections proven to be caused by methicillin-resistant *Staphylococcus aureus*. Am J Surg 2010; 199: 804–16. 20227056 10.1016/j.amjsurg.2009.08.045

[23] Talan DA , Lovecchio F, Abrahamian FM et al A randomized trial of clindamycin versus trimethoprim-sulfamethoxazole for uncomplicated wound infection. Clin Infect Dis 2016; 62: 1505–13. 27025829 10.1093/cid/ciw177 PMC4885652

[24] Corey GR , Wilcox M, Talbot GH et al Integrated analysis of CANVAS 1 and 2: phase 3, multicenter, randomized, double-blind studies to evaluate the safety and efficacy of ceftaroline versus vancomycin plus aztreonam in complicated skin and skin-structure infection. Clin Infect Dis 2010; 51: 641–50. 20695801 10.1086/655827

[25] Noel GJ , Strauss RS, Amsler K et al Results of a double-blind, randomized trial of ceftobiprole treatment of complicated skin and skin structure infections caused by Gram-positive bacteria. Antimicrob Agents Chemother 2008; 52: 37–44. 17954698 10.1128/AAC.00551-07 PMC2223887

[26] Noel GJ , Bush K, Bagchi P et al A randomized, double-blind trial comparing ceftobiprole medocaril with vancomycin plus ceftazidime for the treatment of patients with complicated skin and skin-structure infections. Clin Infect Dis 2008; 46: 647–55. 18225981 10.1086/526527

[27] Boucher HW , Wilcox M, Talbot GH et al Once-weekly dalbavancin versus daily conventional therapy for skin infection. N Engl J Med 2014; 370: 2169–79. 24897082 10.1056/NEJMoa1310480

[28] Corey GR , Arhin FF, Wikler MA et al Pooled analysis of single-dose oritavancin in the treatment of acute bacterial skin and skin-structure infections caused by Gram-positive pathogens, including a large patient subset with methicillin-resistant *Staphylococcus aureus*. Int J Antimicrob Agents 2016; 48: 528–34. 27665522 10.1016/j.ijantimicag.2016.07.019

[29] Corey GR , Good S, Jiang H et al Single-dose oritavancin versus 7-10 days of vancomycin in the treatment of Gram-positive acute bacterial skin and skin structure infections: the SOLO II noninferiority study. Clin Infect Dis 2015; 60: 254–62. 25294250 10.1093/cid/ciu778

[30] Corey GR , Kabler H, Mehra P et al Single-dose oritavancin in the treatment of acute bacterial skin infections. N Engl J Med 2014; 370: 2180–90. 24897083 10.1056/NEJMoa1310422

[31] Stryjewski ME , Graham DR, Wilson SE et al Telavancin versus vancomycin for the treatment of complicated skin and skin-structure infections caused by Gram-positive organisms. Clin Infect Dis 2008; 46: 1683–93. 18444791 10.1086/587896

[32] Moran GJ , Fang E, Corey GR et al Tedizolid for 6 days versus linezolid for 10 days for acute bacterial skin and skin-structure infections (ESTABLISH-2): a randomised, double-blind, phase 3, non-inferiority trial. Lancet Infect Dis 2014; 14: 696–705. 24909499 10.1016/S1473-3099(14)70737-6

[33] Prokocimer P , De Anda C, Fang E et al Tedizolid phosphate vs linezolid for treatment of acute bacterial skin and skin structure infections: the ESTABLISH-1 randomized trial. Jama 2013; 309: 559–69. 23403680 10.1001/jama.2013.241

[34] Prince WT , Ivezic-Schoenfeld Z, Lell C et al Phase II clinical study of BC-3781, a pleuromutilin antibiotic, in treatment of patients with acute bacterial skin and skin structure infections. Antimicrob Agents Chemother 2013; 57: 2087–94. 23422913 10.1128/AAC.02106-12 PMC3632892

[35] Pullman J , Gardovskis J, Farley B et al Efficacy and safety of delafloxacin compared with vancomycin plus aztreonam for acute bacterial skin and skin structure infections: a Phase 3, double-blind, randomized study. J Antimicrob Chemother 2017; 72: 3471–80. 29029278 10.1093/jac/dkx329 PMC5890686

[36] Kingsley J , Mehra P, Lawrence LE et al A randomized, double-blind, Phase 2 study to evaluate subjective and objective outcomes in patients with acute bacterial skin and skin structure infections treated with delafloxacin, linezolid or vancomycin. J Antimicrob Chemother 2016; 71: 821–9. 26679243 10.1093/jac/dkv411 PMC4743703

[37] O'Riordan W , Mehra P, Manos P et al A randomized phase 2 study comparing two doses of delafloxacin with tigecycline in adults with complicated skin and skin-structure infections. Int J Infect Dis 2015; 30: 67–73. 25448332 10.1016/j.ijid.2014.10.009

[38] Holland TL , Riordan WO, McManus A et al A phase 3, randomized, double-blind, multicenter study to evaluate the safety and efficacy of intravenous iclaprim versus vancomycin for treatment of acute bacterial skin and skin structure infections suspected or confirmed to be due to Gram-positive pathogens (REVIVE-2 study). Antimicrob Agents Chemother 2018; 62: e02580–17. 29530858 10.1128/AAC.02580-17 PMC5923167

[39] Huang DB , O'Riordan W, Overcash JS et al A Phase 3, Randomized, Double-Blind, Multicenter Study to Evaluate the Safety and Efficacy of Intravenous Iclaprim Vs Vancomycin for the Treatment of Acute Bacterial Skin and Skin Structure Infections Suspected or Confirmed to be Due to Gram-Positive Pathogens: REVIVE-1. Clin Infect Dis 2018; 66: 1222–9. 29281036 10.1093/cid/cix987

[40] Paul M , Bishara J, Yahav D et al Trimethoprim-sulfamethoxazole versus vancomycin for severe infections caused by meticillin resistant *Staphylococcus aureus*: randomised controlled trial. Bmj 2015; 350: h2219. 25977146 10.1136/bmj.h2219 PMC4431679

[41] Davis J , Sud A, O'Sullivan MVN et al Combination of Vancomycin and β-Lactam Therapy for Methicillin-Resistant *Staphylococcus aureus* Bacteremia: A Pilot Multicenter Randomized Controlled Trial. Clin Infect Dis 2016; 62: 173–80. 26349552 10.1093/cid/civ808

[42] Rehm SJ , Boucher H, Levine D et al Daptomycin versus vancomycin plus gentamicin for treatment of bacteraemia and endocarditis due to *Staphylococcus aureus*: subset analysis of patients infected with methicillin-resistant isolates. J Antimicrob Chemother 2008; 62: 1413–21. 18782781 10.1093/jac/dkn372 PMC2583068

[43] Thwaites GE , Scarborough M, Szubert A et al Adjunctive rifampicin for *Staphylococcus aureus* bacteraemia (ARREST): a multicentre, randomised, double-blind, placebo-controlled trial. Lancet 2018; 391: 668–78. 29249276 10.1016/S0140-6736(17)32456-X PMC5820409

[44] Davis JS , Van Hal S, Tong SY. Combination antibiotic treatment of serious methicillin-resistant *Staphylococcus aureus* infections. Semin Respir Crit Care Med 2015; 36: 3–16. 25643267 10.1055/s-0034-1396906

[45] Dilworth TJ , Ibrahim O, Hall P et al β-Lactams enhance vancomycin activity against methicillin-resistant *Staphylococcus aureus* bacteremia compared to vancomycin alone. Antimicrob Agents Chemother 2014; 58: 102–9. 24145519 10.1128/AAC.01204-13 PMC3910806

[46] Gould FK , Denning DW, Elliott TS et al Guidelines for the diagnosis and antibiotic treatment of endocarditis in adults: a report of the Working Party of the British Society for Antimicrobial Chemotherapy. J Antimicrob Chemother 2012; 67: 269–89. 22086858 10.1093/jac/dkr450

[47] Wunderink RG , Niederman MS, Kollef MH et al Linezolid in methicillin-resistant *Staphylococcus aureus* nosocomial pneumonia: a randomized, controlled study. Clin Infect Dis 2012; 54: 621–9. 22247123 10.1093/cid/cir895

[48] Rubinstein E , Lalani T, Corey GR et al Telavancin versus vancomycin for hospital-acquired pneumonia due to Gram-positive pathogens. Clin Infect Dis 2011; 52: 31–40. 21148517 10.1093/cid/ciq031 PMC3060890

[49] Awad SS , Rodriguez AH, Chuang YC et al A phase 3 randomized double-blind comparison of ceftobiprole medocaril versus ceftazidime plus linezolid for the treatment of hospital-acquired pneumonia. Clin Infect Dis 2014; 59: 51–61. 24723282 10.1093/cid/ciu219 PMC4305133

[50] Silverman JA , Mortin LI, Vanpraagh AD et al Inhibition of daptomycin by pulmonary surfactant: *in vitro* modeling and clinical impact. J Infect Dis 2005; 191: 2149–52. 15898002 10.1086/430352

[51] Moher D , Liberati A, Tetzlaff J et al Preferred reporting items for systematic reviews and meta-analyses: the PRISMA statement. PLoS Med 2009; 6: e1000097. 19621072 10.1371/journal.pmed.1000097 PMC2707599

