# Supplementary material for: Treatment of methicillin-resistant Staphylococcus aureus (MRSA): updated guidelines from the UK
Source: JAC Antimicrob Resist. 2021 Feb 3;3(1):dlaa114. doi: 10.1093/jacamr/dlaa114 (PMC8210269; doi:10.1093/jacamr/dlaa114)
Supplement: dlaa114_Supplementary_Data [file dlaa114_supplementary_data.zip › 20-153-Supplementary_data.docx]

**Supplementary data**

**Treatment of methicillin-resistant *Staphylococcus aureus* (MRSA): updated guidelines from the UK**

**Table S1.** Search criteria used for evidence identification.

**Table S2**. Summary of studies excluded from review (n=62).

**Table S3**. Summary of studies included for review (n=30).

**Table S4.** List of stakeholders to whom the guidance was sent for consultation.

**Table S5:** Anonymised Consultation Responses (n=31 comments) (comments are recorded as they were returned).

**References**

**Table S1.** Search criteria used for evidence identification.

| **Search Engine** | **#** | **Search term** |
| --- | --- | --- |
| Cochrane Library | 1 | MeSH descriptor: [Methicillin-Resistant Staphylococcus aureus] explode all trees |
|  | 2 | (((Methicillin or Meticillin) near/2 Resist* near/2 (Staph* or S? aureus)) or MRSA):ti,ab |
|  | 3 | Combine #1 or #2 |
|  | 4 | MeSH descriptor: [Staphylococcus aureus] this term only |
|  | 5 | MeSH descriptor: [Methicillin Resistance] explode all trees |
|  | 6 | Combine #4 and #5 |
|  | 7 | Combine #3 or #6 Publication Year from 2007 |
| EMBASE | 1 | Methicillin Resistant Staphylococcus aureus/ OR (((Methicillin ORMeticillin) adj2 Resist* adj2 (Staph* OR S? aureus)) OR MRSA).ti,ab. |
|  | 2 | Crossover-Procedure/ OR Double-Blind Procedure/ OR Randomized Controlled Trial/ OR Single-Blind Procedure/ OR (Random* OR Factorial* OR Crossover* OR (Cross Over*) OR Cross-Over* OR Placebo* OR (Doubl* adj Blind*) OR (Singl* adj Blind*) OR Assign* OR Allocat* OR Volunteer*).mp. OR ("Interrupted Time Series" OR "ITS Studies" OR "ITS Study" OR "Controlled Before After" OR "Controlled Before and After" OR "CBA Studies" OR "CBA Study").ti,ab. |
|  | 3 | Combine 1 AND 2 |
|  | 4 | Exp Animals/ OR Exp Invertebrate/ OR Animal Experiment/ OR Animal Model/ OR Animal Tissue/ OR Animal Cell/ OR Nonhuman/ |
|  | 5 | Human/ OR Normal Human/ OR Human Cell/ |
|  | 6 | Combine 4 AND 5 |
|  | 7 | Combine 4 NOT 6 |
|  | 8 | Combine 3 NOT 7 |
|  | 9 | Limit 8 to (Exclude Medline Journals AND YR="2007 -Current") |
| MEDLINE | 1 | Methicillin-Resistant Staphylococcus aureus/ OR (Exp Staphylococcus aureus/ AND Exp Methicillin Resistance/) OR (((Methicillin ORMeticillin) adj2 Resist* adj2 (Staph* OR S? aureus)) OR MRSA).ti,ab. |
|  | 2 | Interrupted Time Series Analysis/ OR Controlled Before-After Studies/ OR ("Interrupted Time Series" OR "ITS Studies" OR "ITS Study" OR "Controlled Before After" OR "Controlled Before and After" OR "CBA Studies" OR "CBA Study").ti,ab. OR (Randomized Controlled Trial OR Controlled Clinical Trial OR Pragmatic Clinical Trial).pt. OR (Randomi?ed OR Randomly OR Placebo OR Trial OR Groups).ab. OR Drug Therapy.fs. NOT (Animals NOT (Humans AND Animals)).sh. |
|  | 3 | Combine 1 AND 2 |
|  | 4 | Limit 3 to YR="2007 -Current" |

**Table S2**. Summary of studies excluded from review (n=62).

| **Infection Type** | **Population** | **Intervention** | **Comparator** | **Reason for Exclusion** | **Reference** |
| --- | --- | --- | --- | --- | --- |
| Impetigo | Paediatric | Co-trimoxazole (4mg/kg plus 20mg/kg per dose) q12h for 5 days | Single dose benzathine benzylpenicillin (45 mg/kg up to 900mg) | Less than 10 participants infected with MRSA in each group | Tong *et al.* 2010^52^ |
| Impetigo | Paediatric | Co-trimoxazole (4mg/kg plus 20mg/kg per dose) q12h for 3 days  Co-trimoxazole (8 mg/kg plus 40mg/kg per dose) q24h for 5 days | Benzathine benzylpenicillin (weight-banded IM injection) | Cannot calculate number of patients with MRSA | Bowen *et al.* 2014^53^ |
| Impetigo | Paediatric | Topical minocycline  (1% or 4%) | Topical minocycline  (1% or 4%) | No comparator agent | Chamny *et al.* 2016^54^ |
| Abscess | Adult | Cephalexin 500mg q6h | Placebo | Intervention and comparator are both placebo for MRSA | Rajendran *et al.* 2007^55^ |
| Abscess | Paediatric | Co-trimoxazole  10-12mg/kg/day | Placebo | No MRSA subgroup analysis | Duong *et al.* 2010^56^ |
| Abscess | Adult | Co-trimoxazole  320mg/1600mg q12h | Placebo | No MRSA subgroup analysis | Schmitz *et al.* 2010^57^ |
| Abscess | ≥12 years | Co-trimoxazole  320mg/1600mg q12h | Placebo | No MRSA subgroup analysis | Talan *et al.* 2016^21^ |
| SSTI | Adult  Paediatric | Cephalexin 500mg q6h plus co-trimoxazole 160mg/800mg to 320mg/1600mg q12h | Cephalexin 500mg q6h plus placebo | Less than 10 participants infected with MRSA in each group | Pallin *et al.* 2013^58^ |
| SSTI | ≥12 years | Cephalexin 500mg q6h plus co-trimoxazole  320mg/1600mg q12h | Cephalexin 500mg q6h plus placebo | Data not interpretable: unclear about number of MRSA at the beginning of study | Moran *et al.* 2017^59^ |
| SSTI | Adult | Ceftaroline 600 mg q12h | Vancomycin 1g q12h | Less than 10 participants infected with MRSA in each group | Talbot *et al.* 2007^60^ |
| SSTI | Adult | Ceftaroline (600 mg q8h) | Vancomycin (15 mg/kg q12h) plus aztreonam (1g q8h) | Less than 50 participants infected with MRSA in each group | Dryden *et al.* 2016^61^ |
| SSTI | Paediatric | Ceftaroline (dose adjusted for age and weight) | Vancomycin or cefazolin, plus optional aztreonam | Less than 10 participants infected with MRSA in each group | Korczowski *et al.* 2016^62^ |
| SSTI | Paediatric | Ceftriaxone 50 mg/kg once daily via OPAT | Hospitalised: IV flucloxacillin 50 mg/kg every 6 hours | Less than 10 participants infected with MRSA in each group | Ibrahim *et al.* 2016^63^ |
| SSTI | Adult | JNJ-Q2 (novel fluoroquinolone) 250mg q12h | Linezolid 600mg q12h | Less than 50 participants infected with MRSA in each group | Covington *et al.* 2011^64^ |
| SSTI | Adult | Dalbavancin 1500mg single dose | Dalbavancin 1000mg dose followed by 500mg 7 days later | No comparator agent | Dunne *et al.* 2016^65^ |
| SSTI | Adult | Oritavancin Daily dose 200mg for 3-7 days; single 1200mg dose or 800mg dose with option for 400mg on day 5 | Oritavancin Daily dose 200mg for 3-7 days; single 1200mg dose or 800mg dose with option for 400mg on day 5 | No comparator agent | Dunbar *et al*. 2011^66^ |
| SSTI | Adult | Oritavancin  1200mg q24h | Vancomycin 1g q12h | Further analysis of SOLO-I and SOLO-2 studies (already included) | Lodise *et al.* 2017^67^ |
| SSTI | Adult | Telavancin 10mg/kg/day | Vancomycin 1g q12h | Subset analysis of the telavancin ATLAS studies (already included) | Wilson *et al.* 2009^68^ |
| SSTI | Adult | TD-1792 (cefilavancin) 2mg/kg q24h | Vancomycin 1g q12h | Less than 50 participants infected with MRSA in each group | Strykewski *et al.* 2012^69^ |
| SSTI | Adult  Paediatric | Clindamycin 300mg q8h | Co-trimoxazole 320mg/1600mg q12h | No MRSA subgroup analysis | Miller *et al.* 2015^70^ |
| SSTI | Adult | Omadacycline 100mg q24h | Linezolid 600mg q12h | Less than 50 participants infected with MRSA in each group | Noel *et al.* 2012^71^ |
| SSTI  Diabetic foot | Adult | Linezolid 600mg q12h  (oral or IV) | Vancomycin 1g q12h | Pooled results of three previously published RCTS | Lipsky *et al.* 2011^72^ |
| SSTI | Adult | Linezolid 600mg q12h | Vancomycin 1g q12h | Less than 50 participants infected with MRSA in each group | McCollum *et al.* 2007^73^ |
| SSTI | Adult | Linezolid 600mg q12h | Vancomycin 1g q12h | Based on Weigelt *et al.* 2005,^74^ included in previous guideline | McKinnon *et al.* 2006^75^ |
| SSTI | Adult | Tedizolid 200mg od | Linezolid 600mg q12h | No MRSA subgroup analysis | Joseph *et al.* 2017^76^ |
| SSTI | Adult | Tedizolid 200mg q24h | Linezolid 600mg q12h | Less than 50 participants infected with MRSA in each group | Mikamo *et al.* 2018^77^ |
| SSTI | Adult | Daptomycin 10mg/kg/day | Vancomycin 1g q12h | Less than 50 participants infected with MRSA in each group | Katz *et al.* 2008^78^ |
| SSTI | Adult | Daptomycin 4mg/kg/day | Vancomycin | Less than 50 participants infected with MRSA in each group | Kauf *et al.* 2015^79^ |
| SSTI | Paediatric | Daptomycin once daily at age-dependent doses | Standard of care | No MRSA subgroup analysis | Bradley *et al.* 2017^80^ |
| SSTI | Adult | Daptomycin 4mg/kg/day | Vancomycin 1g q12h | Bias in study design (4:1 daptomycin: vancomycin ratio); less than 50 participants infected with MRSA in vancomycin group | Aikawa *et al.* 2013^81^ |
| SSTI | Adult | Gepotidacin: 750mg q12h; 1000mg q12h, or 1000mg q8h | Gepotidacin: 750mg q12h; 1000mg q12h, or 1000mgq8h | No comparator agent | O'Riordan *et al.* 2017^82^ |
| SSTI | Adult | Iclaprim 0.8 mg/kg body weight 1.6 mg/kg body weight | Vancomycin 1g q12h | Less than 10 participants infected with MRSA in each group | Krievins *et al.* 2009^83^ |
| SSTI, CR-BSI | ≥ 13 years, ≥40kg | Linezolid 600mg q12h | Vancomycin 1g q12h | Less than 50 participants infected with MRSA in each group | Wilcox *et al.* 2009^84^ |
| Bone | Adult | Daptomycin 6mg/kg/day q24h | Standard therapy  [Either vancomycin (1g q12h with appropriate dose adjustment) or an anti-staphylococcal penicillin (nafcillin, oxacillin or flucloxacillin) 2g q4h) depending on the susceptibility of the causative strain] | Less than 10 participants infected with MRSA in each group | Lalani *et al.* 2008^85^ |
| Bacteraemia | Adult | Telavancin 10 mg/kg q24h | Vancomycin 1g q12h or anti-staphylococcal penicillin (nafcillin 2g q6h, oxacillin 2g q6h, or cloxacillin 2 g q6h) | Less than 10 participants infected with MRSA in each group | Strykewski *et al.* 2014^86^ |
| Bacteraemia | Adult | Daptomycin 6 mg/kg body weight of daptomycin infused over 30 min every 24 h | Vancomycin 15 mg/kg body weight of vancomycin infused every 12 h over 2 h with appropriate dose adjustments | Less than 10 participants infected with MRSA in each group | Kalimuddin *et al.* 2018^87^ |
| Bacteraemia,  endocarditis | Adult | Daptomycin 6mg/kg q24h (participants with left-sided endocarditis also received 1mg/kg gentamicin q8h) | Low-dose gentamicin (1mg/kg q8h) plus either an anti-staphylococcal penicillin (nafcillin, oxacillin, or flucloxacillin 2g q6h) or vancomycin 1g q12h | Less than 50 participants infected with MRSA in each group | Fowler *et al.* 2006^88^ |
| SSTI or pneumonia | Adult | Linezolid 600mg q12h | Vancomycin 1g q12h | Less than 50 participants infected with MRSA in each group | Lin *et al.* 2008^89^ |
| CAP | Adult | Ceftaroline 600mg q12h | Ceftriaxone 1g q24h | Integrated analysis of FOCUS trials. Less than 10 participants infected with MRSA in each group | File *et al.* 2010^90^ |
| CAP | Adult | Ceftaroline 600mg q12h | Ceftriaxone 1g q24h | Retrospective analysis of FOCUS trials | Eckberg *et al.* 2012^91^ |
| CAP | Adult | Ceftaroline 600mg q12h | Ceftriaxone 2g q24h | Low numbers of *S. aureus* included and no MRSA subgroup analysis | Zhong *et al*. 2015^92^ |
| CAP | Paediatric | Ceftaroline (dose adjusted for age and weight) | Ceftriaxone with vancomycin (dose adjusted for age and weight) | Less than 10 participants infected with MRSA in each group | Blumer *et al.* 2016^93^ |
| CAP | Paediatric | Ceftaroline (dose adjusted for age and weight) | Ceftriaxone (dose adjusted for age and weight) | Less than 10 participants infected with MRSA in each group | Cannavino *et al.* 2016^94^ |
| CAP | Adult | Ceftobiprole 500mg with placebo | Ceftriaxone 2g 30 min infusion with linezolid 600mg q12h | Less than 10 participants infected with MRSA in each group | Nicholson *et al.* 2012^95^ |
| CAP | Adult | Solithromycin 800mg day 1, 1400mg days 2-5, placebo on days 6-7 | Moxifloxacin 400mg q24h days 1-7 | No MRSA subgroup analysis | Barrera *et al.*^96^ |
| CAP | Adult | Solithromycin 400mg q24h IV, switched to oral at investigator’s discretion  The initial oral dose of solithromycin was 800mg; subsequent oral doses were 400 mg once daily. | Moxifloxacin 400mg q24h (IV) switched to 400mg q24h oral at investigator’s discretion | Less than 10 participants infected with MRSA in each group | File *et al.* 2016^97^ |
| CAP | Adult | Nemonoxacin 500mg q24h  Nemonoxacin 500mg q24h | Levofloxacin 500mg q24h | Less than 10 participants infected with MRSA in each group | Liu *et al.* 2015^98^ |
| HAP | Adult | Vancomycin 1g q12h with rifampicin 300mg q12h | Vancomycin 1g q12h | Less than 50 participants infected with MRSA in each group | Jung *et al.* 2010^99^ |
| HAP | Adult | Linezolid 600mg q12h | Vancomycin 1g q12h | Less than 50 participants infected with MRSA in each group | Wunderink *et al.* 2008^100^ |
| HAP | Adult | Linezolid 600mg q12h | Vancomycin 15 mg/kg per dose | Economic analysis of Wunderink *et al.,* 2012^47^ No new efficacy data | Rello *et al.* 2016^101^ |
| HAP | Adult | Linezolid 600mg q12h | Vancomycin 1g q12h | Reassessment of Wunderink *et al.* 2012^47^ Assessment of renal function; no new efficacy data | Liu *et al.* 2017^102^ |
| Cystic fibrosis | Adults | Nebulized vancomycin  (250 mg in 5mL sterile water, twice per day) | Nebulised placebo (quinine 0.1 mg/mL in 5mL sterile water, twice per day) | Colonisation of CF participants - not treatment | Dezube *et al.* 2018^103^ |
| Cystic fibrosis | Ages 4-45 years | Oral co-trimoxazole, or if sulfa-allergic, minocycline plus oral rifampicin; chlorhexidine mouthwash for 2 weeks; nasal mupirocin and chlorhexidine body wipes for 5 days and environmental decontamination for 21 days. | Observation only - No antibiotics or other intervention | Colonisation of CF participants - not treatment | Muhlebach *et al.* 2017^104^ |
| Various | ≥13 years,  ≥40kg Neutropenic participants with cancer | Linezolid 600mg q12h | Vancomycin 1g q12h | Less than 10 participants infected with MRSA in each group | Jaksic *et al.* 2006^105^ |
| SSTI, BJI, pneumonia, bacteraemia | Adult | Co-trimoxazole 160mg/800mg q8h with rifampicin 600mg q24h | Linezolid 600mg q12h | Less than 50 participants infected with MRSA in each group | Harbath *et al.* 2014^106^ |
| Pneumonia, SSTI and sepsis | Not stated | Linezolid 600mg q12h | Vancomycin 1g q12h | Less than 50 participants infected with MRSA in each group | Kohno *et al.* 2007^107^ |
| Pneumonia, SSTI, sepsis | Not stated | Linezolid 600mg q12h | Vancomycin 1g q12h | Less than 10 participants infected with MRSA in each group | Jindal *et al*. 2015^108^ |
| Prophylaxis for cardiac surgery | Adult | Cefuroxime 1.5g | Vancomycin 1g | Prophylaxis | Garey *et al.* 2008^109^ |
| Prophylaxis for vascular surgery | Adult | Cefazolin with vancomycin | Cefazolin plus daptomycin | Prophylaxis | Stone *et al.* 2010^110^ |
| Prophylaxis for vascular surgery | Adult | Cefazolin  Weight-based dosing | Cefazolin with vancomycin  Cefazolin with daptomycin  Weight-based dosing | Prophylaxis | Stone *et al.* 2015^111^ |
| Prophylaxis for cerebral shunt placement | Adult | Cefazolin 1.5g | Vancomycin 1g | Prophylaxis | Tacconelli *et al.* 2008^112^ |
| Prophylaxis for primary hip and knee arthroplasty | Adult | Cefuroxine 1.5g (3 doses) | Fusidic acid 500mg (3 doses)  Vancomycin 500mg (3 doses) | Prophylaxis | Tyllianakis *et al*. 2010^113^ |

Key: Adult defined as ≥18 years; CAP, community-acquired pneumonia; CR-BSI, catheter-related bloodstream infection; CF, cystic fibrosis; CAP, community-acquired pneumonia; HAP, hospital-acquired pneumonia; IM, intramuscular; OPAT, outpatient parenteral antimicrobial therapy; SSTI, skin and soft tissue infection.

**References cited in Table S2** (citations follow on from those citations included in the main document)

**52.** Tong SY, Andrews RM, Kearns T *et al.* Trimethopim-sulfamethoxazole compared with benzathine penicillin for treatment of impetigo in Aboriginal children: a pilot randomised controlled trial. *J Paediatr Child Health* 2010; **46**: 131-3.

**53.** Bowen AC, Tong SY, Andrews RM *et al.* Short-course oral co-trimoxazole versus intramuscular benzathine benzylpenicillin for impetigo in a highly endemic region: an open-label, randomised, controlled, non-inferiority trial. *Lancet* 2014; **384**: 2132-40.

**54.** Chamny S, Miron D, Lumelsky N *et al.* Topical Minocycline Foam for the Treatment of Impetigo in Children: Results of a Randomized, Double-Blind, Phase 2 Study. *J Drugs Dermatol* 2016; **15**: 1238-43.

**55.** Rajendran PM, Young D, Maurer T *et al.* Randomized, double-blind, placebo-controlled trial of cephalexin for treatment of uncomplicated skin abscesses in a population at risk for community-acquired methicillin-resistant *Staphylococcus aureus* infection. *Antimicrob Agents Chemother* 2007; **51**: 4044-8.

**56.** Duong M, Markwell S, Peter J *et al.* Randomized, controlled trial of antibiotics in the management of community-acquired skin abscesses in the pediatric patient. *Ann Emerg Med* 2010; **55**: 401-7.

**57.** Schmitz GR, Bruner D, Pitotti R *et al.* Randomized controlled trial of trimethoprim-sulfamethoxazole for uncomplicated skin abscesses in patients at risk for community-associated methicillin-resistant *Staphylococcus aureus* infection. *Ann Emerg Med* 2010; **56**: 283-7.

**21.** Talan DA, Mower WR, Krishnadasan A *et al.* Trimethoprim-Sulfamethoxazole versus Placebo for Uncomplicated Skin Abscess. *N Engl J Med* 2016; **374**: 823-32.

**58.** Pallin DJ, Binder WD, Allen MB *et al.* Clinical trial: comparative effectiveness of cephalexin plus trimethoprim-sulfamethoxazole versus cephalexin alone for treatment of uncomplicated cellulitis: a randomized controlled trial. *Clin Infect Dis* 2013; **56**: 1754-62.

**59.** Moran GJ, Krishnadasan A, Mower WR *et al.* Effect of cephalexin plus trimethoprim-sulfamethoxazole vs cephalexin alone on clinical cure of uncomplicated cellulitis: A randomized clinical trial. *JAMA* 2017; **317**: 2088-96.

**60.** Talbot GH, Thye D, Das A *et al.* Phase 2 study of ceftaroline versus standard therapy in treatment of complicated skin and skin structure infections. *Antimicrob Agents Chemother* 2007; **51**: 3612-6.

**61.** Dryden M, Zhang Y, Wilson D *et al.* A Phase III, randomized, controlled, non-inferiority trial of ceftaroline fosamil 600 mg every 8 h versus vancomycin plus aztreonam in patients with complicated skin and soft tissue infection with systemic inflammatory response or underlying comorbidities. *J Antimicrob Chemother* 2016; **71**: 3575-84.

**62.** Korczowski B, Antadze T, Giorgobiani M *et al.* A Multicenter, Randomized, Observer-blinded, Active-controlled Study to Evaluate the Safety and Efficacy of Ceftaroline Versus Comparator in Pediatric Patients With Acute Bacterial Skin and Skin Structure Infection*. Pediatr Infect Dis J* 2016; **35**: e239-47.

**63.** Ibrahim LF, Hopper SM, Babl FE *et al.* Who can have parenteral antibiotics at home? A prospective observational study in children with moderate/severe cellulitis. *Pediatr Infect Dis J* 2016; **35**: 269‐74.

**64.** Covington P, Davenport JM, Andrae D *et al.* Randomized, double-blind, phase II, multicenter study evaluating the safety/tolerability and efficacy of JNJ-Q2, a novel fluoroquinolone, compared with linezolid for treatment of acute bacterial skin and skin structure infection. *Antimicrob Agents Chemother* 2011; **55**: 5790-7.

**65.** Dunne MW, Puttagunta S, Giordano P *et al.* A Randomized Clinical Trial of Single-Dose Versus Weekly Dalbavancin for Treatment of Acute Bacterial Skin and Skin Structure Infection. *Clin Infect Dis* 2016; **62**: 545-51.

**66.** Dunbar LM, Milata J, McClure T *et al.* Comparison of the efficacy and safety of oritavancin front-loaded dosing regimens to daily dosing: an analysis of the SIMPLIFI trial. *Antimicrob Agents Chemother* 2011; **55**: 3476-84.

**67.** Lodise TP, Redell M, Armstrong SO *et al.* Efficacy and Safety of Oritavancin Relative to Vancomycin for Patients with Acute Bacterial Skin and Skin Structure Infections (ABSSSI) in the Outpatient Setting: Results From the SOLO Clinical Trials. *Open Forum Infect Dis* 2017; **4**: ofw274.

**68.** Wilson SE, O'Riordan W, Hopkins A *et al.* Telavancin versus vancomycin for the treatment of complicated skin and skin-structure infections associated with surgical procedures. *Am J Surg* 2009; **197**: 791-6.

**69.** Stryjewski ME, Potgieter PD, Li YP *et al.* TD-1792 versus vancomycin for treatment of complicated skin and skin structure infections. *Antimicrob Agents Chemother* 2012; **56**: 5476-83.

**70.** Miller LG, Daum RS, Creech CB *et al.* Clindamycin versus trimethoprim-sulfamethoxazole for uncomplicated skin infections. *N Engl J Med* 2015; **372**: 1093-103.

**71.** Noel GJ, Draper MP, Hait H *et al.* A randomized, evaluator-blind, phase 2 study comparing the safety and efficacy of omadacycline to those of linezolid for treatment of complicated skin and skin structure infections. *Antimicrob Agents Chemother* 2012; **56**: 5650-4.

**72.** Lipsky BA, Itani KM, Weigelt JA *et al.* The role of diabetes mellitus in the treatment of skin and skin structure infections caused by methicillin-resistant *Staphylococcus aureus*: results from three randomized controlled trials. *Int J Infect Dis* 2011; **15**: e140-6.

**73.** McCollum M, Sorensen SV, Liu LZ. A comparison of costs and hospital length of stay associated with intravenous/oral linezolid or intravenous vancomycin treatment of complicated skin and soft-tissue infections caused by suspected or confirmed methicillin-resistant *Staphylococcus aureus* in elderly US patients. *Clin Ther* 2007; **29**: 469-77.

**74.** Weigelt J, Itani K, Stevens D *et al.* Linezolid versus vancomycin in treatment of complicated skin and soft tissue infections. *Antimicrob Agents Chemother* 2005; **49**: 2260-6.

**75.** McKinnon PS, Sorensen SV, Liu LZ *et al.* Impact of linezolid on economic outcomes and determinants of cost in a clinical trial evaluating patients with MRSA complicated skin and soft-tissue infections. *Ann Pharmacother* 2006; **40**: 1017-23.

**76.** Joseph WS, Culshaw D, Anuskiewicz S *et al.* Tedizolid and Linezolid for Treatment of Acute Bacterial Skin and Skin Structure Infections of the Lower Extremity versus Non-Lower-Extremity Infections Pooled Analysis of Two Phase 3 Trials. *J Am Podiatric Med Assoc* 2017; **107**: 264-71.

**77.** Mikamo H, Takesue Y, Iwamoto Y *et al.* Efficacy, safety and pharmacokinetics of tedizolid versus linezolid in patients with skin and soft tissue infections in Japan - Results of a randomised, multicentre phase 3 study. *J Infect Chemother* 2018; **24**: 434-42.

**78.** Katz DE, Lindfield KC, Steenbergen JN *et al.* A pilot study of high-dose short duration daptomycin for the treatment of patients with complicated skin and skin structure infections caused by Gram-positive bacteria. *Int J Clin Pract* 2008; **62**: 1455-64.

**79.** Kauf TL, McKinnon P, Corey GR *et al.* An open-label, pragmatic, randomized controlled clinical trial to evaluate the comparative effectiveness of daptomycin versus vancomycin for the treatment of complicated skin and skin structure infection. *BMC Infect Dis* 2015; **15**: 503.

**80.** Bradley J, Glasser C, Patino H *et al.* Daptomycin for complicated skin infections: A randomized trial. *Pediatrics* 2017; **139**: e20162477.

**81.** Aikawa N, Kusachi S, Mikamo H *et al.* Efficacy and safety of intravenous daptomycin in Japanese patients with skin and soft tissue infections. *J Infect Chemother* 2013; **19**: 447-55.

**82.** O'Riordan W, Tiffany C, Scangarella-Oman N *et al.* Efficacy, safety, and tolerability of gepotidacin (GSK2140944) in the treatment of patients with suspected or confirmed Gram-positive acute bacterial skin and skin structure infections. *Antimicrob Agents Chemother* 2017; **61**: e02095-16.

**83.** Krievins D, Brandt R, Hawser S *et al.* Multicenter, randomized study of the efficacy and safety of intravenous iclaprim in complicated skin and skin structure infections. *Antimicrob Agents Chemother* 2009; **53**: 2834-40.

**84.** Wilcox MH, Tack KJ, Bouza E *et al.* Complicated skin and skin-structure infections and catheter-related bloodstream infections: noninferiority of linezolid in a phase 3 study. *Clin Infect Dis* 2009; **48**: 203-12.

**85.** Lalani T, Boucher HW, Cosgrove SE *et al.* Outcomes with daptomycin versus standard therapy for osteoarticular infections associated with *Staphylococcus aureus* bacteraemia. *J Antimicrob Chemother* 2008; **61**: 177-82.

**86.** Stryjewski ME, Lentnek A, O'Riordan W *et al.* A randomized Phase 2 trial of telavancin versus standard therapy in patients with uncomplicated *Staphylococcus aureus* bacteremia: the ASSURE study. *BMC Infect Dis* 2014; **14**: 289.

**87.** Kalimuddin S, Chan YFZ, Phillips R *et al.* A randomized phase 2B trial of vancomycin versus daptomycin for the treatment of methicillin-resistant *Staphylococcus aureus* bacteremia due to isolates with high vancomycin minimum inhibitory concentrations - results of a prematurely terminated study. *Trials* 2018; **19**: 305.

**88.** Fowler VG, Jr., Boucher HW, Corey GR *et al.* Daptomycin versus standard therapy for bacteremia and endocarditis caused by *Staphylococcus aureus*. *N Engl J Med* 2006; **355**: 653-65.

**89.** Lin DF, Zhang YY, Wu JF *et al.* Linezolid for the treatment of infections caused by Gram-positive pathogens in China. *Int J Antimicrob Agents* 2008; **32**: 241-9.

**90.** File TM, Jr., Low DE, Eckburg PB *et al.* Integrated analysis of FOCUS 1 and FOCUS 2: randomized, doubled-blinded, multicenter phase 3 trials of the efficacy and safety of ceftaroline fosamil versus ceftriaxone in patients with community-acquired pneumonia. *Clin Infect Dis* 2010; **51**: 1395-405.

**91.** Eckburg P, Friedland D, Llorens L *et al.* Day 4 clinical response of ceftaroline fosamil versus cefriaxone for community-acquired bacterial pneumonia. *Infect Dis Clin Pract* 2012; **20**: 254-60.

**92.** Zhong NS, Sun T, Zhuo C *et al.* Ceftaroline fosamil versus ceftriaxone for the treatment of Asian patients with community-acquired pneumonia: a randomised, controlled, double-blind, phase 3, non-inferiority with nested superiority trial. *Lancet Infect Dis* 2015; **15**: 161-71.

**93.** Blumer JL, Ghonghadze T, Cannavino C *et al.* A Multicenter, Randomized, Observer-blinded, Active-controlled Study Evaluating the Safety and Effectiveness of Ceftaroline Compared With Ceftriaxone Plus Vancomycin in Pediatric Patients With Complicated Community-acquired Bacterial Pneumonia. *Pediatr Infect Dis J* 2016; **35**: 760-6.

**94.** Cannavino CR, Nemeth A, Korczowski B *et al.* A Randomized, Prospective Study of Pediatric Patients With Community-acquired Pneumonia Treated With Ceftaroline Versus Ceftriaxone. *Pediatr Infect Dis J* 2016; **35**: 752-9.

**95.** Nicholson SC, Welte T, File TM, Jr. *et al.* A randomised, double-blind trial comparing ceftobiprole medocaril with ceftriaxone with or without linezolid for the treatment of patients with community-acquired pneumonia requiring hospitalisation. *Int J Antimicrob Agents* 2012; **39**: 240-6.

**96.** Barrera CM, Mykietiuk A, Metev H *et al.* Efficacy and safety of oral solithromycin versus oral moxifloxacin for treatment of community-acquired bacterial pneumonia: a global, double-blind, multicentre, randomised, active-controlled, non-inferiority trial (SOLITAIRE-ORAL). *Lancet Infect Dis* 2016; **16**: 421-30.

**97.** File TM, Jr., Rewerska B, Vucinic-Mihailovic V *et al.* SOLITAIRE-IV: A Randomized, Double-Blind, Multicenter Study Comparing the Efficacy and Safety of Intravenous-to-Oral Solithromycin to Intravenous-to-Oral Moxifloxacin for Treatment of Community-Acquired Bacterial Pneumonia. *Clin Infect Dis* 2016; **63**: 1007-16.

**98.** Liu Y, Zhang Y, Wu J *et al.* A randomized, double-blind, multicenter Phase II study comparing the efficacy and safety of oral nemonoxacin with oral levofloxacin in the treatment of community-acquired pneumonia. *J Microbiol Immunol Infect* 2017; **50**: 811-20.

**99.** Jung YJ, Koh Y, Hong SB *et al.* Effect of vancomycin plus rifampicin in the treatment of nosocomial methicillin-resistant *Staphylococcus aureus* pneumonia. *Crit Care Med* 2010; **38**: 175-80.

**100.** Wunderink RG, Mendelson MH, Somero MS *et al.* Early microbiological response to linezolid vs vancomycin in ventilator-associated pneumonia due to methicillin-resistant *Staphylococcus aureus*. *Chest* 2008; **134**: 1200-7.

**101.** Rello J, Nieto M, Sole-Violan J *et al.* Nosocomial pneumonia caused by methicillin-resistant *Staphylococcus aureus* treated with linezolid or vancomycin: A secondary economic analysis of resource use from a Spanish perspective. *Medicina Intensiva* 2016; **40**: 474-82.

**102.** Liu P, Capitano B, Stein A *et al.* Clinical outcomes of linezolid and vancomycin in patients with nosocomial pneumonia caused by methicillin-resistant *Staphylococcus aureus* stratified by baseline renal function: a retrospective, cohort analysis. *BMC Nephrology* 2017; **18**: 168.

**103.** Dezube R, Jennings MT, Rykiel M *et al.* Eradication of persistent methicillin-resistant *Staphylococcus aureus* infection in cystic fibrosis. *J Cyst Fibros* 2018; <http://dx.doi.org/10.1016/j.jcf.2018.07.005>.

**104.** Muhlebach MS, Beckett V, Popowitch E *et al.* Microbiological efficacy of early MRSA treatment in cystic fibrosis in a randomised controlled trial. *Thorax* 2017; **72**: 318-26.

**105.** Jaksic B, Martinelli G, Perez-Oteyza J *et al.* Efficacy and safety of linezolid compared with vancomycin in a randomized, double-blind study of febrile neutropenic patients with cancer. *Clin Infect Dis* 2006; **42**: 597-607.

**106.** Harbarth S, von Dach E, Pagani L *et al.* Randomized non-inferiority trial to compare trimethoprim/sulfamethoxazole plus rifampicin versus linezolid for the treatment of MRSA infection. *J Antimicrob Chemother* 2015; **70**: 264-72.

**107.** Kohno S, Yamaguchi K, Aikawa N *et al.* Linezolid versus vancomycin for the treatment of infections caused by methicillin-resistant *Staphylococcus aureus* in Japan. *J Antimicrob Chemother* 2007; **60**: 1361-9.

**108.** Jindal NJ, S; Maitra, D. Efficacy of linezolid over vancomycin in treating methicillin resistant *Staphylococcus aureus* (MRSA) infections. *Int J Pharma Bio Sci* 2015; **6**: 1087-95.

**109.** Garey KW, Lai D, Dao-Tran TK *et al.* Interrupted time series analysis of vancomycin compared to cefuroxime for surgical prophylaxis in patients undergoing cardiac surgery. *Antimicrob Agents Chemother* 2008; **52**: 446-51.

**110.** Stone PAC, J; AbuRahma, A; Safley, L; Emmett, M; Asmita M. Vascular Surgical Antibiotic Prophylaxis Study (VSAPS). *Vasc Endovasc Surg* 2010; **44**: 521-8.

**111.** Stone PA, AbuRahma AF, Campbell JR *et al.* Prospective randomized double-blinded trial comparing 2 anti-MRSA agents with supplemental coverage of cefazolin before lower extremity revascularization. *Ann Surg* 2015; **262**: 495-501.

**112.** Tacconelli E, Cataldo MA, Albanese A *et al.* Vancomycin versus cefazolin prophylaxis for cerebrospinal shunt placement in a hospital with a high prevalence of meticillin-resistant *Staphylococcus aureus*. *J Hosp Infect* 2008; **69**: 337-44.

**113.** Tyllianakis ME, Karageorgos A, Marangos MN *et al.* Antibiotic prophylaxis in primary hip and knee arthroplasty: comparison between cefuroxime and two specific antistaphylococcal agents. *J Arthroplasty* 2010; **25**: 1078-82.

**Table S3**. Summary of studies included for review (n=30).

| **Infection Type** | **Population** | **Intervention**  **Outcome** | **Comparator**  **Outcome** | **Primary Test/**  **Assessment of Cure** | **Conclusion**  **(as stated in the reference)** | **Treatment Difference**  **(95% CI intervals)  [p value]** | **Reference** |
| --- | --- | --- | --- | --- | --- | --- | --- |
| Traumatic lesions and impetigo | >2 months | Retapamulin ointment (1%) q12h plus oral placebo  Clinical success: 39/61 (63.9%) | Linezolid q12h-q8h plus placebo ointment  Clinical success: 29/32 (90.6%) | 7-9 days after therapy | Clinical success rate at follow-up in the per-protocol MRSA population was significantly lower in the retapamulin versus the linezolid group | -26.7  (-45.7, -7.7)  PP-MRSA group | Tanus *et al.* 2014^15^ |
| Abscess | Adult/Paediatric | Incision/drainage plus clindamycin 300mg q8h  Cure rate ITT:  116/142 (81.7%)  Incision/drainage plus co-trimoxazole (160:800mg bd plus placebo for midday dose)  Cure rate ITTP:  110/130 (84.6%) | Incision/drainage plus placebo  Cure rate ITT:  73/116 (62.9%) | End of treatment | As compared with incision and drainage alone, clindamycin or co-trimoxazole in conjunction with incision and drainage improved short-term outcomes in patients who have a simple abscess | Clindamycin vs. co-trimoxazole (p=0.63)  Placebo vs. clindamycin (p<0.001)  Placebo vs. co-trimoxazole  (p = 0.001) | Daum *et al.* 2017^18^ |
| Abscess | ≥12 years | Co-trimoxazole (7-days (4 single-strength pills, 80 mg/400 mg each, twice daily)  Clinical cure:  203/219 (92.7%) | Placebo (4 pills twice daily)  Clinical cure: 202/249 (81.1%) | 7-14 days after treatment | Treatment of abscesses with co-trimoxazole after incision and drainage may be of benefit in patients with MRSA infection | 11.6 (5.2, 18.0) | Talan *et al.* 2018^20^ |
| Surgical drainage of abscess | Paediatric | Co-trimoxazole 3/7 10 mg/kg per day divided twice a day  Treatment failure:  8/69 (12%)  Recurrent infection: 8/60 (13%) | Co-trimoxazole 10/7 10 mg/kg per day divided twice a day  Treatment failure:  1/69 (1%)  Recurrent infection: 2/67 (3%) | 10-14 days after treatment | Patients with MRSA skin abscesses are more likely to experience treatment failure and recurrent skin infection if given 3 rather than 10 days of co-trimoxazole after surgical drainage | Treatment Failure:  10.1, (12.1, 18.2) [p=0.03] Recurrent infection: 10.3 (0.8, 19.9) [p=0.046] | Holmes *et al.* 2016^19^ |
| SSTI | Paediatric | Cephalexin 40mg/kg/day  6/64 (9%) primary infection worsened | Clindamycin 20mg/kg/day  2/71 (3%) primary infection worsened | 48-72 hours | There was no significant difference between cephalexin and clindamycin for treatment of uncomplicated paediatric SSTI caused predominantly by CA-MRSA | [p=0.15] | Chen *et al.* 2011^17^ |
| SSTI | Adult | Ceftaroline  600mg q12h  Clinical cure mMITT: 155/179 (86.6%) | Vancomycin 1g q12h + Aztreonam 1g q12h  Clinical cure mMITT: 124/151 (82.1%) | 8-15 days after treatment | Ceftaroline achieved high clinical cure rates, was efficacious against cSSSI caused by MRSA and other common cSSSI pathogens | No values | Corey *et al.* 2010^24^ |
| SSTI | Adult | Ceftobiprole  500mg q12h  Clinical cure ME: 56/61 (91.8%) | Vancomycin 1g q12h  Clinical cure ME: 54/60 (90%) | 10-14 days after treatment | There was no statistically significant difference in outcome between the two groups of patients infected with MRSA | 1.8%  (-8.4, 12.1) | Noel *et al.* 2008^25^ |
| SSTI | Adult | Ceftobiprole  500mg for 120 mins q8h plus placebo  Clinical cure CE:  78/87 (89.7%) | Vancomycin 1g q12h plus ceftazidime  Clinical cure CE:  31/36 (86.1%) | 7-14 days after treatment | In patients with MRSA, clinical cure rates were similar between the two treatment arms | 3.6%  (-8.0 to 19.7) | Noel *et al.* 2008^26^ |
| SSTI | Adult | Delafloxacin 300mg q12h  Clinical cure: 13/14 (92.9%)  Delafloxacin 45mg q12h  Clinical cure:  19/20 (95% | Tigecycline 100mg once, 50mg q12h  Clinical cure:  12/14 (85.7%) | 14-21 days after treatment | Delafloxacin was similarly effective as tigecycline for a variety of complicated skin and skin-structure infections and was well tolerated | No significant difference between treatment arms | O’Riordan *et al*. 2015^37^ |
| SSTI | Adult | Delafloxacin 300 mg q12h  Clinical cure MITT:  19/29 (65.5%) | Linezolid 600 mg q12h  Clinical cure MITT:  21/34 (61.8%)  Vancomycin 15 mg/kg (actual body weight) q12h  Clinical cure MITT:  21/32 (65.6%) | 48-72 hours | There was no statistically significant difference in outcome between the three groups of patients | No values | Kingsley *et al*. 2016^36^ |
| SSTI | Adult | Delafloxacin 300mg  Objective response:  190/220 (86.4%) | Vancomycin 15mg/kg q12h plus aztreonam 2g q12h  Objective response:  199/225 (88.4%) | 48-72 hours | There was no statistically significant difference in outcome between the two groups of patients infected with MRSA | -2.0 (-8.4, 4.16) | Pullman *et al.* 2017^35^ |
| SSTI | Adult | Dalbavancin 1g (d1), 500mg (d8)  Treatment success: 72/74 (97.3%) | Vancomycin 15 mg/kg bd (at least 3 days) followed by oral linezolid 600mg q12h  Treatment success: 49/50 (98%) | 48-72 hours | Once-weekly IV dalbavancin was not inferior to twice-daily IV vancomycin followed by oral linezolid for the treatment of ABSSSI caused by MRSA | No values | Boucher *et al.* 2014^27^ |
| SSTI | Adult | Oritavancin  1200mg q24h  Primary efficacy outcome at ECE:  84/104 (80.8%) | Vancomycin 1g q12h  Primary efficacy outcome at ECE: 80/100 (80%) | 48-72 hours | A single dose of oritavancin was not inferior to twice-daily vancomycin administered for 7 to 10 days for the treatment of ABSSSI caused by MRSA | 0.8  (-10.1, 11.7) | Corey *et al.* 2014^30^ |
| SSTI | Adult | Oritavancin  1200mg q24h  Primary efficacy outcome at ECE: 82/100 (82%) | Vancomycin 1g q12h  Primary efficacy outcome at ECE: 82/101 (82.2%) | 48-72 hours | A single 1200-mg dose of oritavancin was not inferior to 7–10 days of vancomycin in treating ABSSSI caused by MRSA | 0.8  (-9.9, 11.5) | Corey *et al.* 2015^29^ |
| SSTI | Adult | Oritavancin  1200mg q24h  Primary efficacy outcome at ECE: 166/204 (81.4%) | Vancomycin 1g q12h  Primary efficacy outcome at ECE: 162/201 (80.6%) | 48-72 hours | A single 1200-mg dose of oritavancin was not inferior to 7–10 days of vancomycin in treating ABSSSI caused by MRSA | 0.8 (-6.9 to 8.4) | Corey *et al.* 2016^28^ |
| SSTI | Adult | Telavancin 10mg/kg/day  Clinical cure ME: 252/278 (90.6%) | Vancomycin 1g q12h  Clinical cure ME: 260/301 (86.4%) | 10-14 days after treatment | Telavancin given once daily is at least as effective as vancomycin for the treatment of patients with cSSTI, including those infected with MRSA | 4.1  (-1.1, 9.3) | Strykewski *et al.* 2008^31^ |
| SSTI | ≥12 years | Clindamycin (7-day course of clindamycin (one 300-mg capsule, 4 times daily, with 3 placebo capsules, twice daily for first and third doses)  Clinical cure PP: 70/78 (89.7%) | Co-trimoxazole (4 single-strength capsules, 80 mg/400 mg, twice daily, with 1 placebo capsule, twice daily for second and fourth doses)  Clinical cure PP: 78/83 (94%) | 7-14 days after treatment | In settings where MRSA is prevalent, clindamycin and co-trimoxazole produce similar cure and adverse event rates among patients with an uncomplicated wound infection | 4.2 (-13.9, 5.5) | Talan *et al.* 2016^23^ |
| SSTI | Adult | Linezolid 600mg q12h (oral or IV)  Clinical success at end of study PP:  191/227 (84%) | Vancomycin 15 mg/kg twice daily  Clinical success at end of study PP:  167/209 (80%) | 7-10 days after treatment | Linezolid is an effective alternative to vancomycin for the treatment of cSSTl caused by MRSA | [p=0.249] | Itani *et al.* 2010^22^ |
| SSTI | Adult | Tedizolid 200mg q24h  Primary efficacy outcome at ECE:  75/88 (85.2%) | Linezolid 600mg q12h  Primary efficacy outcome at ECE:  77/90 (85.6%) | 48-72 hours | Tedizolid was a statistically noninferior treatment to linezolid in early clinical response at 48 to 72 hours after initiating therapy for an ABSSSI. | No value | Prokocimer *et al.* 2013^33^ |
| SSTI | ≥12 years | Tedizolid 200mg q24h  Clinical response:  44/53 (83%) | Linezolid 600mg q12h  Clinical response:  44/56 (79%) | 48-72 hours | Intravenous to oral once-daily tedizolid 200mg for 6 days was non-inferior to twice-daily linezolid 600mg for 10 days for treatment of patients with ABSSSI caused by MRSA | 4·4  (–10·8 to 19·5) | Moran *et al*. 2014^32^ |
| SSTI | Adult | Iclaprim 80mg q12h  Clinical response: 61/69 (88.4%) | Vancomycin  15mg/kg q12h  Clinical Response: 53/69 (73.8%) | 48-72 hours | Iclaprim achieved non-inferiority compared with vancomycin at its primary endpoint of early clinical response | 11.6  (-5.80, 28.48) | Holland *et al.* 2018^38^ |
| SSTI | Adult | Iclaprim 80mg bd  Clinical Response: 59/73 (80.8%) | Vancomycin  15mg/kg q12h  Clinical Response: 50/61 (82%) | 48-72 hours | Iclaprim achieved non-inferiority compared with vancomycin at its primary endpoint of early clinical response | –1.15  (–17.9, 15.8) | Huang *et al*. 2018^39^ |
| SSTI | Adult | BC-3781 (Lefamulin)  100mg or 150mg q12h  Clinical success: 100mg: 29/34 pts (85.3%)  150mg: 28/32 pts (87.5%) | Vancomycin 1g q12h  Clinical success: 32/39 (82.1%) | 10-14 days after treatment | Clinical cure rates were similar in all three groups of patients with MRSA | No values | Prince *et al*. 2013^34^ |
| HAP | Adult | Telavancin 10mg/kg/day  Clinical cure: 104/139 (74.8%) | Vancomycin 1g q12h  Clinical cure: 115/154 (74.7%) | 7-14 days after treatment | Telavancin is noninferior to vancomycin on the basis of clinical response in the treatment of HAP caused by MRSA | 0.4 (–9.5, 10.4) | Rubenstein *et al.* 2011^48^ |
| HAP | Adult | Linezolid 600mg q12h  Clinical success PP: 95/165 (57.6%) | Vancomycin 15mg/kg q12h  Clinical success PP: 81/174 (46.6%) | End of study | For the treatment of MRSA nosocomial pneumonia, clinical response at end of study in the per-protocol population was significantly higher with linezolid than with vancomycin, although 60-day mortality was similar | 11 (0.5, 21.6)  [p=0.042] | Wunderink *et al.* 2012^47^ |
| HAP | Adult | Ceftobiprole 500mg q8h  Clinical cure ITT:  22/28 (78.6%) | Ceftazidime 2g q8h plus linezolid 600mg q12h  Clinical cure ITT:  19/32 (59.4%) | 7–14 days | Ceftobiprole is non inferior to the combination of ceftazidime and linezolid | 19.2  (−3.6 to 42.0) | Awad *et al*. 2014^49^ |
| Bacteraemia | Adult | Standard therapy plus rifampicin (600mg or 900mg)  Treatment failure: 9/26 (34.6%) | Standard therapy plus placebo  Treatment failure: 3/21 (14.3%) | Time to bacteriologically confirmed treatment failure, disease recurrence or death | Adjunctive rifampicin provided no overall benefit over standard antibiotic therapy in adults with *S. aureus* bacteraemia | 2.74  (0.74, 10.15) | Thwaites *et al.* 2018^43^ |
| Bacteraemia | Adult | Vancomycin 1.5g q12h  29 patients ITT Mean duration of bacteraemia 3.00 (3.35) | Vancomycin 1.5g q12h plus flucloxacillin 2g q6h  31 patients ITT Mean duration of bacteraemia 1.94 (1.79) | Duration of MRSA bacteraemia (days) | Combining an anti-staphylococcal β-lactam with vancomycin may shorten the duration of MRSA bacteraemia | 0.65  (0.41, 1.02)  [p=0.06] | Davis *et al.* 2016^41^ |
| Bacteraemia (uncomplicated/complicated)  Endocarditis | Adult | Daptomycin 6mg/kg q24h  Treatment success:  Uncomplicated B  6/10 (60%)  Complicated B:  10/22 (45%)  Right sided IE:  4/8 (50%) | Vancomycin1g q12h with gentamicin 1mg/kg q8h (for the first 4 days of treatment)  Treatment success:  5/11 (45%)  Complicated B  6/22 (27%)  Right sided IE  3/6 (50%) | Clinical and bacteriological recovery 6 weeks after end of treatment | Daptomycin was an effective alternative to vancomycin plus low dose gentamicin for MRSA uncomplicated bacteraemia or right-sided endocarditis. | 11.9  (28.3 to 32.1) | Rehm *et al.* 2008^42^ |
| SSTI, BJI, Endovascular, pneumonia, bacteraemia, other | Adult | Co-trimoxazole 320/1600 q12h  Treatment failure at day 7  BJI: 11/39 (28%)  B: 20/50 (40%) | Vancomycin 1g bdq12h  Treatment failure at day 7  BJI: 7/32 (22%)  B: 23/41 (56%) | Treatment failure at 7 days | High dose co-trimoxazole did not achieve non-inferiority to vancomycin in the treatment of severe MRSA infections. The difference was particularly marked for patients with bacteraemia | B: 1.4  (0.91, 2.16) | Paul *et al.* 2015^40^ |

Key: Adult defined as ≥18 years; ABSSSI, acute bacterial skin and skin structure infection; B, bacteraemia, BJI, bone and joint infection; CAP, community-acquired pneumonia; CE, clinically evaluable population; CR-BSI, catheter-related bloodstream infection; cSSSI, complicated skin and skin structure infection; CF, cystic fibrosis; ECE, early clinical evaluation; HAP, hospital-acquired pneumonia; IE, infective endocarditis; ME, microbiologically evaluable population; mMITT, microbiological modified intent-to-treat; OPAT, outpatient parenteral antimicrobial therapy; PP, per-protocol population, SSTI, skin and soft tissue infection. *Clinical/Treatment success is defined as clinical cure plus improved.

**References cited in Table S3** (each citation remains the same as the main guideline document)

**15.** Tanus T, Scangarella-Oman NE, Dalessandro M *et al.* A randomized, double-blind, comparative study to assess the safety and efficacy of topical retapamulin ointment 1% versus oral linezolid in the treatment of secondarily infected traumatic lesions and impetigo due to methicillin-resistant *Staphylococcus aureus*. *Adv Skin Wound Care* 2014; **27**: 548-59.

**17.** Chen AE, Carroll KC, Diener-West M *et al.* Randomized controlled trial of cephalexin versus clindamycin for uncomplicated pediatric skin infections. *Pediatrics* 2011; **127**: e573-80.

**18.** Daum RS, Miller LG, Immergluck L *et al.* A placebo-controlled trial of antibiotics for smaller skin abscesses. *N Engl J Med* 2017; **376**: 2545-55.

**19.** Holmes L, Ma C, Qiao H *et al.* Trimethoprim-Sulfamethoxazole Therapy Reduces Failure and Recurrence in Methicillin-Resistant *Staphylococcus aureus* Skin Abscesses after Surgical Drainage. *J Pediatr* 2016; **169**: 128-34 e1.

**20.** Talan DA, Moran GJ, Krishnadasan A *et al.* Subgroup Analysis of Antibiotic Treatment for Skin Abscesses. *Ann Emerg Med* 2018; **71**: 21-30.

**22.** Itani KM, Dryden MS, Bhattacharyya H *et al.* Efficacy and safety of linezolid versus vancomycin for the treatment of complicated skin and soft-tissue infections proven to be caused by methicillin-resistant *Staphylococcus aureus*. *Am J Surg* 2010; **199**: 804-16.

**23.** Talan DA, Lovecchio F, Abrahamian FM *et al.* A Randomized Trial of Clindamycin Versus Trimethoprim-sulfamethoxazole for Uncomplicated Wound Infection. *Clin Infect Dis* 2016; **62**: 1505-13.

**24.** Corey GR, Wilcox M, Talbot GH *et al.* Integrated analysis of CANVAS 1 and 2: phase 3, multicenter, randomized, double-blind studies to evaluate the safety and efficacy of ceftaroline versus vancomycin plus aztreonam in complicated skin and skin-structure infection. *Clin Infect Dis* 2010; **51**: 641-50.

**25.** Noel GJ, Strauss RS, Amsler K *et al.* Results of a double-blind, randomized trial of ceftobiprole treatment of complicated skin and skin structure infections caused by Gram-positive bacteria. *Antimicrob Agents Chemother* 2008; **52**: 37-44.

**26.** Noel GJ, Bush K, Bagchi P *et al.* A randomized, double-blind trial comparing ceftobiprole medocaril with vancomycin plus ceftazidime for the treatment of patients with complicated skin and skin-structure infections. *Clin Infect Dis* 2008; **46**: 647-55.

**27.** Boucher HW, Wilcox M, Talbot GH *et al.* Once-weekly dalbavancin versus daily conventional therapy for skin infection. *N Engl J Med* 2014; **370**: 2169-79.

**28.** Corey GR, Arhin FF, Wikler MA *et al.* Pooled analysis of single-dose oritavancin in the treatment of acute bacterial skin and skin-structure infections caused by Gram-positive pathogens, including a large patient subset with methicillin-resistant *Staphylococcus aureus*. *Int J Antimicrob Agents* 2016; **48**: 528-34.

**29.** Corey GR, Good S, Jiang H *et al.* Single-dose oritavancin versus 7-10 days of vancomycin in the treatment of Gram-positive acute bacterial skin and skin structure infections: the SOLO II noninferiority study. *Clin Infect Dis* 2015; **60**: 254-62.

**30.** Corey GR, Kabler H, Mehra P *et al.* Single-dose oritavancin in the treatment of acute bacterial skin infections. *N Engl J Med* 2014; **370**: 2180-90.

**31.** Stryjewski ME, Graham DR, Wilson SE *et al.* Telavancin versus vancomycin for the treatment of complicated skin and skin-structure infections caused by Gram-positive organisms. *Clin Infect Dis* 2008; **46**: 1683-93.

**32.** Moran GJ, Fang E, Corey GR *et al.* Tedizolid for 6 days versus linezolid for 10 days for acute bacterial skin and skin-structure infections (ESTABLISH-2): a randomised, double-blind, phase 3, non-inferiority trial. *Lancet Infect Dis* 2014; **14**: 696-705.

**33.** Prokocimer P, De Anda C, Fang E *et al.* Tedizolid phosphate vs linezolid for treatment of acute bacterial skin and skin structure infections: the ESTABLISH-1 randomized trial. *JAMA* 2013; **309**: 559-69.

**34.** Prince WT, Ivezic-Schoenfeld Z, Lell C *et al.* Phase II clinical study of BC-3781, a pleuromutilin antibiotic, in treatment of patients with acute bacterial skin and skin structure infections. *Antimicrob Agents Chemother* 2013; **57**: 2087-94.

**35.** Pullman J, Gardovskis J, Farley B *et al.* Efficacy and safety of delafloxacin compared with vancomycin plus aztreonam for acute bacterial skin and skin structure infections: a Phase 3, double-blind, randomized study. *J Antimicrob Chemother* 2017; **72**: 3471-80.

**36.** Kingsley J, Mehra P, Lawrence LE *et al.* A randomized, double-blind, Phase 2 study to evaluate subjective and objective outcomes in patients with acute bacterial skin and skin structure infections treated with delafloxacin, linezolid or vancomycin. *J Antimicrob Chemother* 2016; **71**: 821-9.

**37.** O'Riordan W, Mehra P, Manos P *et al.* A randomized phase 2 study comparing two doses of delafloxacin with tigecycline in adults with complicated skin and skin-structure infections. *Int J Infect Dis* 2015; **30**: 67-73.

**38.** Holland TL, Riordan WO, McManus A *et al.* A phase 3, randomized, double-blind, multicenter study to evaluate the safety and efficacy of intravenous iclaprim versus vancomycin for treatment of acute bacterial skin and skin structure infections suspected or confirmed to be due to Gram-positive pathogens (REVIVE-2 study). *Antimicrob Agents Chemother* 2018; **62**: e02580.

**39.** Huang DB, O'Riordan W, Overcash JS *et al.* A Phase 3, Randomized, Double-Blind, Multicenter Study to Evaluate the Safety and Efficacy of Intravenous Iclaprim Vs Vancomycin for the Treatment of Acute Bacterial Skin and Skin Structure Infections Suspected or Confirmed to be Due to Gram-Positive Pathogens: REVIVE-1. *Clin Infect Dis* 2018; **66**: 1222-9.

**40.** Paul M, Bishara J, Yahav D *et al.* Trimethoprim-sulfamethoxazole versus vancomycin for severe infections caused by meticillin resistant *Staphylococcus aureus*: randomised controlled trial. *BMJ* 2015; **350**: h2219.

**41.** Davis J, Sud A, O'Sullivan MVN *et al.* Combination of Vancomycin and beta-Lactam Therapy for Methicillin-Resistant *Staphylococcus aureus* Bacteremia: A Pilot Multicenter Randomized Controlled Trial. *Clin Infect Dis* 2016; **62**: 173-80.

**42.** Rehm SJ, Boucher H, Levine D *et al.* Daptomycin versus vancomycin plus gentamicin for treatment of bacteraemia and endocarditis due to *Staphylococcus aureus*: subset analysis of patients infected with methicillin-resistant isolates. *J Antimicrob Chemother* 2008; **62**: 1413-21.

**43.** Thwaites GE, Scarborough M, Szubert A *et al.* Adjunctive rifampicin for *Staphylococcus aureus* bacteraemia (ARREST): a multicentre, randomised, double-blind, placebo-controlled trial. *Lancet* 2018; **391**: 668-78.

**47.** Wunderink RG, Niederman MS, Kollef MH *et al.* Linezolid in methicillin-resistant *Staphylococcus aureus* nosocomial pneumonia: a randomized, controlled study. *Clin Infect Dis* 2012; **54**: 621-9.

**48.** Rubinstein E, Lalani T, Corey GR *et al.* Telavancin versus vancomycin for hospital-acquired pneumonia due to Gram-positive pathogens. *Clin Infect Dis* 2011; **52**: 31-40.

**49.** Awad SS, Rodriguez AH, Chuang YC *et al.* A phase 3 randomized double-blind comparison of ceftobiprole medocaril versus ceftazidime plus linezolid for the treatment of hospital-acquired pneumonia. *Clin Infect Dis* 2014; **59**: 51-61.

**Table S4.** List of stakeholders to whom the guidance was sent for consultation.

|  | **Consultation Group** |
| --- | --- |
| A | Academy of Medical Royal Colleges |
|  | Association for Nurse Prescribing |
|  | Association of Paediatric Emergency Medicine |
|  | Association of Surgeons of Great Britain & Ireland |
|  | Association of the British Pharmaceutical Industry |
| B | British Association for Cancer Surgery |
|  | British Association of General Paediatrics |
|  | British Association of Paediatric Nephrology |
|  | British Association of Paediatric Surgeons |
|  | British Association of Plastic, Reconstructive and Aesthetic Surgeons |
|  | British Cardiac Patients Association |
|  | British Cardiovascular Society |
|  | British Dental Association |
|  | British Heart Foundation |
|  | British Heart Rhythm Society (formerly Heart Rhythm UK) |
|  | British Heart Valve Society |
|  | British HIV Association |
|  | British Infection Association |
|  | British Lung Foundation |
|  | British Medical Association |
|  | British Orthopaedic Association |
|  | British Paediatric Allergy, Immunology & Infection Group |
|  | British Paediatric Respiratory Society |
|  | British Pharmacological Society |
|  | British Society for Children’s Orthopaedic Surgery |
|  | British Society of Echocardiography |
|  | British Society for Medical Mycology |
|  | British Society for Paediatric Gastroenterology, Hepatology and Nutrition |
| C | Care Quality Commission |
|  | CDiff Support |
|  | Central Sterilising Club |
|  | Children’s Cancer and Leukaemia Group |
|  | Children’s HIV Association |
|  | Clinical Virology Network |
|  | Community Pharmacy Scotland (formerly Scottish Pharmaceutical General Council) |
|  | Consumer Futures (formerly the National Consumer Council) |
| D | Department of Health and Children (Ireland) |
|  | Department of Health Social Services & Public Safety (NHS Northern Ireland) |
| E | European Society of Clinical Microbiology and Infectious Disease |
| F | Faculty of Intensive Care Medicine |
|  | Faculty of Pharmaceutical Medicine |
|  | Faculty of Public Health |
| G | General Dental Council |
|  | General Medical Council |
|  | General Pharmaceutical Council |
|  | Guild of Healthcare Pharmacists |
| H | Health Protection Society |
|  | Health Protection Scotland |
|  | Healthcare Improvement Scotland (NHS) |
|  | Healthcare Infection Society |
|  | Heart Research UK |
| I | Independent Alliance of Patients and Healthcare Workers for Hand Hygiene |
|  | Infection Prevention Society |
|  | Institute of Decontamination Sciences |
| L | Lee Spark Necrotising Fasciitis Foundation |
| M | Medical Defence Union |
|  | Medical Protection Society |
|  | Medical Research Council |
|  | Medical Schools Council |
|  | Microbiology Society |
|  | MRSA Action UK |
| N | National Infusion and Vascular Access Society |
|  | National Institute for Health and Clinical Excellence |
|  | National Institute for Health Research |
|  | National Pharmacy Association |
|  | Neonatal and Paediatric Pharmacists Group |
|  | NHS Commissioning Board Special Health Authority |
|  | NHS Confederation |
|  | NHS England |
|  | NHS Improvement |
|  | NHS Providers |
| P | Parliamentary and Health Service Ombudsman |
|  | Paediatric Intensive Care Society |
|  | Paediatric Microbiology Group |
|  | Patients Association |
|  | Pharmaceutical Quality Group |
|  | Pharmaceutical Society of Northern Ireland |
|  | Public Health England |
|  | Public Health Wales |
|  | **Consultation Group** |
| Q | Quality Improvement Scotland (NHS) |
| R | Research Quality Association |
|  | Royal College of Anaesthetists |
|  | Royal College of Emergency Medicine |
|  | Royal College of General Practitioners |
|  | Royal College of Midwives |
|  | Royal College of Nursing |
|  | Royal College of Obstetricians & Gynaecologists |
|  | Royal College of Ophthalmologists |
|  | Royal College of Pathologists |
|  | Royal College of Paediatrics and Child Health |
|  | Royal College of Physicians & Surgeons |
|  | Royal College of Physicians of London |
|  | Royal College of Psychiatrists |
|  | Royal College of Radiologists |
|  | Royal College of Surgeons |
|  | Royal College of Surgeons (Edinburgh) |
|  | Royal Pharmaceutical Society |
|  | Royal Society for Public Health |
|  | Royal Society of Tropical Medicine and Hygiene |
| S | Scottish Association of Health Councils |
|  | Scottish Intercollegiate Guidelines Network |
|  | Scottish Medicines Consortium |
|  | Society for Acute Medicine |
|  | Society of Critical Care Medicines |
|  | Standards for Microbiology Investigations |
|  | Surviving Sepsis Campaign |
| T | The British Society for Allergy & Clinical Immunology |
|  | The British Thoracic Society |
|  | The Consumers' Association (Which?) |
| U | UK Clinical Pharmacy Association |
| W | Welsh Assembly Government |
|  | Welsh Microbiological Association |

**Table S5:** Anonymised Consultation Responses (n=31 comments) (comments are recorded as they were returned).

| **SOURCE** | **PAGE** | **SECTION, PARAGRAPH,**  **TABLE, RECOMMENDATION** | **COMMENTS** | **COMMITTEE RESPONSE / DECISION  (changes made refer to version180220)** |
| --- | --- | --- | --- | --- |
| Lay representative MRSA Action UK | 1 | 11 | Include “MRSA” in the keywords | ACTION: Added Line 11 |
| Lay representative MRSA Action UK | 3 | 43 (contents page) | Include full description for CNS and bracket acronym | ACTION: Added Line 1074 - will be updated in contents page once document is updated |
| Lay representative MRSA Action UK | 4 | 45 (contents page) | Include full description for CSF and bracket acronym | ACTION: Added Line 1085 - will be updated in contents page once document is updated |
| Lay representative MRSA Action UK | 5 | 61 | The comment on the treatment of sepsis for hospitalised patients needs to be put into context, it doesn’t say why this is significant. | ACTION: the following sentence has been removed at Line 310  "These changes have implications for the empirical treatment of sepsis in hospitalised patients" |
| Lay representative MRSA Action UK | 5 | 61 | There is a reference to sepsis and its impact, but it’s not very well explained in the guideline. I have included something in the lay summary and am assuming this is the context in which it was mentioned | Text reads: “The number of MRSA bloodstream infections in UK hospitals has fallen since 2008, which affects how patients with sepsis, a serious life-threatening infection, are treated. Antibiotics are one of the main treatments for sepsis. Identifying the most appropriate antibiotic and giving them promptly increases the possibility of surviving sepsis, including patients who may have MRSA.” |
| Assistant Director of Nursing, Beaumont Hospital and Royal College of Surgeons in Ireland | 6 | General | Comprehensive management of infection should consider concurrent decolonisation for treatment success and to prevent recurrence. Therefore, recommendation on decolonisation of skin and naso-oro-pharyngeal colonisation would strengthen the guideline and provide an adequate resource for all relevant healthcare professionals. | ACTION: additional text added Line 389 "Recommendations relating to infection prevention and control of MRSA, including decolonisation, are considered in a separate guideline written by HIS and IPS." |
| Lay Representative |  | 60 | ‘less common’ than what? It reads as if serious MRSA infection is less common than the incidence which has fallen. Suggest rephrase for clarity or if you just mean that serious MRSA infections have reduced in number then say this. | ACTION: Removal of the latter part of the sentence (and serious infection caused by MRSA is now less common.)Line 331 |
| Lay Representative |  | 84 | Is another purpose of the guide to alert pts to the current evidence and what they might expect? If so put this in too. | ACTION: none COMMENT: the authors agree that this is not within the scope of the guideline |
| Lay Representative |  | 89 | This might be the place to say that a member of the public reviewed the guidelines. | ACTION: An acknowledgement has been added at Line 347 and text added at line 366: "The guideline was reviewed independently by two lay representatives" |
| Lay representative MRSA Action UK | 6 | 89 | Add “Draft recommendations were written by the guideline development group. These were circulated (17.12.19) to a comprehensive list of stakeholders and uploaded to the BSAC website (www.bsac.org.uk) for a four-week consultation period. Final alterations were made to the document in response to the consultation process.” | ACTION: none COMMENT: this text is provided at Line 538, which is considered the appropriate place by the authors. |
| Lay Representative | 17 | 303 and 306 | 303 & 306 where you recommend that something ‘may be of benefit’ and then ‘should’ might be worth highlighting to demonstrate the strength of the suggestion. | Action: recommendations are being rewritten according to NICE requirements. |
| Lay Representative | 25 | 486(i) | This is an example of no change in the guideline so perhaps flag it as ‘no change’ to existing management at the top. | ACTION: none. COMMENT: The authors have discussed the need to distinguish between existing and new recommendations and agree that for the sake of clarity, the recommendations will not be labelled as 'old/new', rather that the updated guideline will contain all necessary recommendations, past and present. |
| Consultant Microbiologist NHS Lothian | 26 | 574 | Please could put a warning in about drug interactions with rifampicin. Rifampicin is proven to render the following drugs sub therapeutic when used in combination: Linezolid; fusidic acid.  Rifampicin enhances the metabolism of doxycycline; trimethoprim. Please check for drug interactions before using rifampicin as part of combination therapy in patients.  References have been provided within the email text. | ACTION: Text added to Line 395 "Although some guidance may be given as to dosing or drug interactions in particular cases this was not considered to be within the aim of these guidelines and users should seek information on dosing and interactions elsewhere such as in the BNF." |
| Alder Hey Children’s NHS Foundation Trust | 26 | 573-4 | Text says; Therapeutic options include doxycycline, trimethoprim, co-trimoxazole, quinolones, rifampicin, clindamycin, linezolid and co-trimoxazole [Category IA]. Comment - 1. Trimethoprim is not very active against Staph and sensitivity testing may not be available, so would be a poor choice. 2. Cotrimoxazole is included twice | ACTION:  1) Trimethoprim breakpoints are only available for uncomplicated UTI. Reference to this agent in other infections has been removed. 2) duplicate co-trimoxazole has been removed |
| Lay Representative | 29 | 555 | Do you need to say anything about how to prevent SSI rather than just treat it? | ACTION: none COMMENT: prevention of infection (SSTI or bone and joint infection) is not within the scope of this guideline. Prevention of infection will be considered within the HIS/IPS guideline. |
| Lay Representative | 29 | 555 | I thought there was evidence from the Oviva study that oral was as good as IV antibiotics for SSIs? I’m a lay person so I may be completely wrong but I’m mentioning it in case it’s important to check. | ACTION: none COMMENT: the OVIVA study has been considered by the authors; however, the evidence for MRSA was not sufficient for this study to be used as evidence in the present recommendations. |
| Lay Representative | 31  and general | 616 | Where you say something like this about the first line treatment with a particular medication do you need to say anything about the dose, duration and method of delivery? This applies to all recommendations of this type. If it is implied that it is down to personal clinical decision making perhaps the guide should explain that somewhere. | Anna completed ACTION: text added at Line 398 "Although some guidance may be given as to dosing or drug interactions in particular cases this was not considered to be within the aim of these guidelines and users should seek information on dosing and interactions elsewhere such as in the BNF." |
| Alder Hey Children’s NHS Foundation Trust | 29 | 629-30 | Text says; (iv) We recommend a minimum duration of 14 days of antibiotic treatment for patients with uncomplicated MRSA bacteraemia.  Comment This may not apply to children who can be successfully treated with 7 days (Lancet Infect Dis 2016 http://dx.doi.org/10.1016/S1473-3099(16)30024-X) | ACTION: none.  Evidence from the reference listed, specifically the section that refers to S. aureus bacteraeamia, does not support 7 days of antibiotic treatment for children with uncomplicated MRSA bacteraemia:  "In a small trial of neonates with Staphylococcus aureus bacteraemia, Chowdhary and colleagues **showed higher treatment failures with 7 days of intravenous antibiotics than with 14 days.** Three retrospective series documented wide variations in median duration of intravenous antibiotics (5–162 days). In a study of neonates with meticillin-resistant S aureus (MRSA) bacteraemia, the mean duration of vancomycin treatment for those without any complications was 9·7 days (SD 5·1), **although recurrences were greater with fewer than 14 days of antibiotics than with 14 days or longer**.  Children with MRSA bacteraemia without endocarditis had a median antibiotic duration of 22 days (IQR 12–29), with bacteraemia persisting for a median of 6 days (IQR 2–7), despite eﬀective antibiotics." |
| British Thoracic Society | 29 | 651 | Could you add the reasons of withdrawal? “withdrawn due to… and should not…”. | ACTION: text added to Line 1035 "have not been updated since 2008 and we do not recommend that these are used to guide treatment." |
| British Thoracic Society | 30 | 670 | Is it worth including/commenting on the intention to treat analysis? | ACTION: Text deleted at Line 1052 "448 patients (linezolid n=224; vancomycin, n=224) for the modified intention-to-treat analysis and" |
| British Thoracic Society | 31 | 679 | Could you provide examples of the treatment-emergent adverse events? | ACTION: text reworded at Line 1065 "There was an increase in serious adverse events (e.g. septic shock, respiratory failure or multiorgan failure) and treatment-emergent adverse events (e.g. diarrhoea, anaemia, hypokalaemia, constipation, or renal impairment) in patients receiving telavancin (234/751, 31%) compared with those receiving vancomycin (197/752, 26%) overall in this trial (no p value available). |
| British Thoracic Society | 31 | 681 | Is it worth including p value? It seems to be a statistically significant difference but not so strong that it is blindingly obvious by looking at the numbers. |  |
| British Thoracic Society | 31 | 690 | Is the recommendation about glycopeptide or linezolid based on evidence provided in the previous guideline? | ACTION: recommendations has been reworded |
| Lay representative MRSA Action UK | 33 | 693 | Include full description for CNS and bracket acronym | ACTION: Added Line 1074 |
| Lay representative MRSA Action UK | 34 | 704 | Include full description for CSF and bracket acronym | ACTION: Added Line 1085 |
| Lay representative MRSA Action UK |  | Table 1 | Acronym usage – need to include these in a glossary | ACTION: Definitions for acroynms added. COMMENT: The authors agree that once acronyms are fully defined in the text a glossary is not required. |
| Lay Representative |  | General | Somewhere say that the document has been reviewed by a member of the public | ACTION: An acknowledgement has been added at Line 347 and text added at line 366: "The guideline was reviewed independently by two lay representatives" |
| Lay Representative |  | General | It occurs to me that you might want to produce a cut down lay/pt friendly version of the guide to help pts see the current evidence etc this one clearly isn’t and should not be in lay language. | ACTION: none. COMMENT: the guideline is primarily for use by healthcare professionals. A lay summary of the guideline has been provided. |
| Lay Representative |  | General | Where you make specific recommendations e.g. for treatment would it be helpful to say how the new recommendation differs from the old If it does ? or perhaps just clearly flag those that are different so clinicians can see where they need to consider changing practice/treatment. | ACTION: none. COMMENT: The authors have discussed the need to distinguish between existing and new recommendations and agree that for the sake of clarity, the recommendations will not be labelled as 'old/new', rather that the updated guideline will contain all necessary recommendations, past and present. |
| Lay representative MRSA Action UK |  | General | A glossary is needed due to the large number of acronyms used. | ACTION: Definitions for acronyms added. COMMENT: The authors agree that once acronyms are fully defined in the text a glossary is not required. |
| Northern Health and Social Care Trust |  | General | Great work, nothing to add. | ACTION: No response necessary |

-End-
